# Engineering molecular imaging strategies for regenerative medicine

**DOI:** 10.1002/btm2.10114

**Published:** 2018-10-21

**Authors:** Matthew Willadsen, Marc Chaise, Iven Yarovoy, An Qi Zhang, Natesh Parashurama

**Affiliations:** ^1^ Department of Chemical and Biological Engineering University at Buffalo, State University of New York, Furnas Hall Buffalo New York 14228; ^2^ Department of Biomedical Engineering University at Buffalo, State University of New York, Bonner Hall Buffalo New York 14228; ^3^ Jacobs School of Medicine and Biomedical Sciences University at Buffalo State University of New York 955 Main St., Buffalo, New York 14203; ^4^ Clinical and Translation Research Center (CTRC) University at Buffalo, State University of New York 875 Ellicott St., Buffalo, New York 14203

## Abstract

The reshaping of the world's aging population has created an urgent need for therapies for chronic diseases. Regenerative medicine offers a ray of hope, and its complex solutions include material, cellular, or tissue systems. We review basics of regenerative medicine/stem cells and describe how the field of molecular imaging, which is based on quantitative, noninvasive, imaging of biological events in living subjects, can be applied to regenerative medicine in order to interrogate tissues in innovative, informative, and personalized ways. We consider aspects of regenerative medicine for which molecular imaging will benefit. Next, genetic and nanoparticle‐based cell imaging strategies are discussed in detail, with modalities like magnetic resonance imaging, optical imaging (near infra‐red, bioluminescence), raman microscopy, and photoacoustic microscopy), ultrasound, computed tomography, single‐photon computed tomography, and positron emission tomography. We conclude with a discussion of “next generation” molecular imaging strategies, including imaging host tissues prior to cell/tissue transplantation.

**Abbreviations**
ADME: absorption distribution metabolism excretion
ASC: adult stem cell
AuNP: gold (Au) nanoparticle
BLI: bioluminescence imaging
BRET: bioluminescence resonance energy transfer
CAG: chicken beta‐actin/rabbit beta globin hybrid promoter
CAR‐T: chimeric antigen receptor T cell
CCD: charged coupled device
CMV: cytomegalovirus
CSC: cancer stem cell
CT: computed tomography
ESC: embryonic stem cell 18F‐FHBG 9‐(4‐18F‐fluoro‐3‐[hydroxymethyl]butyl)guanine
Fluc: firefly luciferase
Gluc: Gaussia luciferase
GFP: green fluorescent protein
HSC: hematopoietic stem cells
HSV: herpes simplex virus
iPSC: induced pluripotent stem cell
IVM: intravital microscopy
MRI: magnetic resonance imaging
MaSC: mammary stem cells
MSC: mesenchymal stem cell
MPM: multiphoton microscopy
NIR: near infrared
NP: nanoparticle
PA: photoacoustic
PACT: photoacoustic computed tomography
PAM: photoacoustic microscopy
PSC: pluripotent stem cell
PET: positron emission tomography
QD: quantum dot
Rluc: Renilla luciferase
iRFP: bacteria phytochrome photoreceptor iRFP713
RG: reporter gene
SEAP: secreted alkaline phosphatase
SERS: surface‐enhanced Raman scattering
siGNR: single gold nanorod
SPECT: single‐photon emission computer tomography
SPIO: superparamagnetic iron oxide
SWNT: single‐walled nanotube
TSTA: two‐step transcriptional activation
TF: transcription factor
U/S: ultrasound
VEGR: vascular endothelial growth factor receptor

## 1 OVERVIEW

Regenerative medicine is a field that utilizes complex therapies comprised of cells and/or materials, which address failing tissues. Molecular imaging is a branch of radiology that focuses on imaging biology (receptors, biological pathways) rather than anatomy (anatomical imaging like computed tomography [CT] or magnetic resonance imaging [MRI]) or physiology (functional imaging). The goal of molecular imaging is noninvasive imaging, detection, or interrogation of biomolecular events in living subjects, to further understand biology, to detect or diagnose a disease, or to monitor therapy. Molecular imaging has tended to receive more attention in the area of cancer imaging, but how molecular imaging can advance regenerative medicine still needs elucidation. Here, we will review the current state of regenerative medicine and offer new insights into applications of molecular imaging to regenerative medicine. The recurring theme of this review is that merging these regenerative medicine approaches in conjunction with molecular imaging can advance cell therapy in preclinical small animal models, large animal models, and in patients. Furthermore, based on the review these fields, we suggest strategies that will lead to the next generation of regenerative medicine.

## 2 SUMMARY OF KEY CONCEPTS IN REGENERATIVE MEDICINE

Advances in surgery,^1^ like skin grafting,^2^ vascular anastomosis,^3^ and organ transplantation^4^ in part, motivated engineers in the development of artificial organs.^5^ Further advances led to bioartificial organs, tissue engineering and biomaterials,^6^ pluripotent stem cell (PSC) biology,^7^, ^8^ and the first cell therapy using bone marrow.^9^ These various schools of thought share a common goal of treating the patient under conditions of tissue loss or tissue/organ failure. While there has been a focus on various types of impactful therapies, there has been less focus on advancing regenerative medicine through molecular imaging. In the following sections, we define various aspects of regenerative medicine, as they pertain to molecular imaging.

### 2.1 Tissue engineering

Tissue engineering arose in the 1980s as an approach to generate human tissue equivalents for clinical tissue replacement. This creative field encompasses a wide array of approaches and methods involving cell biology, extracellular matrix, and biomimetic material scaffolds. Tissue engineers focused on the transplantation of both cells and scaffolds to reverse tissue/organ failure. In certain cases, the isolation and function of cells were prioritized,^10^ while in other cases, materials design was the major factor that impacted cell and tissue function.^11^ These scaffold‐based approaches involve generating tissue scaffolds using synthetic polymers of various configurations and naturally occurring or engineered biopolymers,^12^ and most recently decellularized scaffolds,^13^ all of which encompass tissue engineering approaches that address tissue loss. As tissues in the body can be broken down into connective tissue, muscle tissue, epithelial tissue, and neural tissue, tissue engineering products can be grouped in this way. Along these lines, tissue engineering strategies have been established for: (a) connective tissues,^14^ including cartilage and bone,^15^ tendons,^16^ and vasculature^17^, ^18^; (b) muscle^19^, ^20^, ^21^; (c) epithelial (internal) organs, including the liver,^22^, ^23^ pancreas,^24^ bladder,^25^ lung,^26^ and kidney^27^; and (d) neural tissue.^28^, ^29^

Upon transplantation of an engineered tissue construct, many critical aspects affect its short‐term and long‐term fate. Vascularization, transport of nutrients and oxygen to the tissue of interest, maintenance of tissue architecture and function, restoration of normal organ function, and integration of the tissue into the whole body are all critical aspects. Conventional imaging can be used to monitor tissue anatomy (i.e., CT for bone regeneration, or MRI for soft tissue regeneration), and functional imaging (i.e., blood flow via MRI or ultrasound [Doppler]). However, another whole dimension of molecular information may be potentially ascertained by applying strategies in molecular imaging to tissue engineering, which could greatly affect outcomes in patients with tissue engineered constructs. These strategies will be further described in section of this review.

### 2.2 Adult (and cancer) stem cells and regenerative biology

In the last 40 years, tremendous efforts in multiple areas of stem cell research have cemented their role in regenerative biology and medicine and helped fortify efforts to translate these findings towards human health. Through techniques developed to isolate adult stem cells (ASC) and assay their capacity for growth and differentiation *in vitro* and *in vivo*, scientists established many fundamental aspects of regenerative biology. Here, we will consider key aspects relevant for application of molecular imaging to ASC and regenerative biology.

ASC are rare (<1%), small, quiescent cells with a high nucleus to cytoplasm ratio. They are central to the tissue generation process by undergoing asymmetric cell divisions into multipotent progenitor cells, which then differentiate into multiple mature cell types. ASC can accomplish this because only ASC, but not their immediate multipotent progenitors, have the capacity to self‐renew. Self‐renewal is a specialized type of cell division that is biologically distinguishable from pure cell division. For example, if ASC divide, they can undergo symmetric self‐renewal divisions into two new ASC, or undergo asymmetric cell divisions into a stem cell and a progenitor cell.

**Figure 1:**
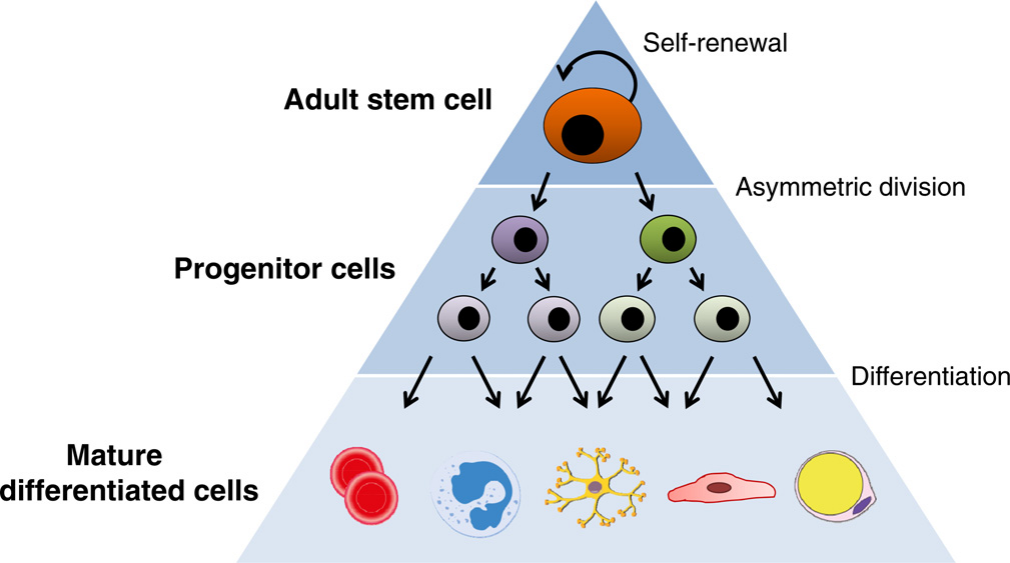
Tissue hierarchy. To maintain tissue indefinitely, ASC undergo asymmetric cell divisions, in which they reform themselves (self‐renewal) and differentiate to give rise to multipotent, and/or committed progenitor cells. These progenitors give rise to mature, differentiated cells, which sit at the bottom of the hierarchy, and provide the bulk of the tissue and organ functions, but have a limited lifespan. When tissues are injured, ASC can increase their activity, to stimulate tissue replacement, sometimes by dividing symmetrically to create more ASC that can replenish tissue

The immediate descendants of ASC are the multipotent progenitor cells, which proliferate and differentiate along different lineages, contributing to tissue homeostasis. These differentiated cells have a limited life span, whereas the ASC, because of their self‐renewal property, have a continuous, unlimited, ability to regenerate themselves. In this way, ASC can both replace themselves and replenish downstream tissues. The corollary of this is that adult, parenchymal tissues are hierarchical with respect to cell type and cell state, and it has been shown that supporting cells can form tissue hierarchies as well.^30^ Taken together, real tissues, as opposed to traditional tissue engineered tissues, are hierarchical and can be visualized as a triangle with horizontal layers (Figure 1). Within this triangle, one or more progenitor cells lie beneath the ASC, and these progenitors, with the appropriate spatiotemporal cues, can proliferate and differentiate into more mature cells. These mature, parenchymal, or functional cells make up the majority of tissue within the organ, and are at the base of the triangle. Two examples include hematopoietic stem cells (HSC),^31^ which give rise to lymphoid versus myeloid lineages in the blood forming system, and mammary stem cells (MaSC), which select between myoepithelial versus luminal lineages in the mammary gland.^32^, ^33^ The activity of these stem cells depends on local or systemic factors, as well as the intrinsic ones, and ultimately the rate of tissue turnover. For example, the intestinal epithelium is renewed at a rate of 3‐5 days, while the blood forming cells are renewed at a rate of ∼25‐50 weeks.^34^

**Figure 2:**
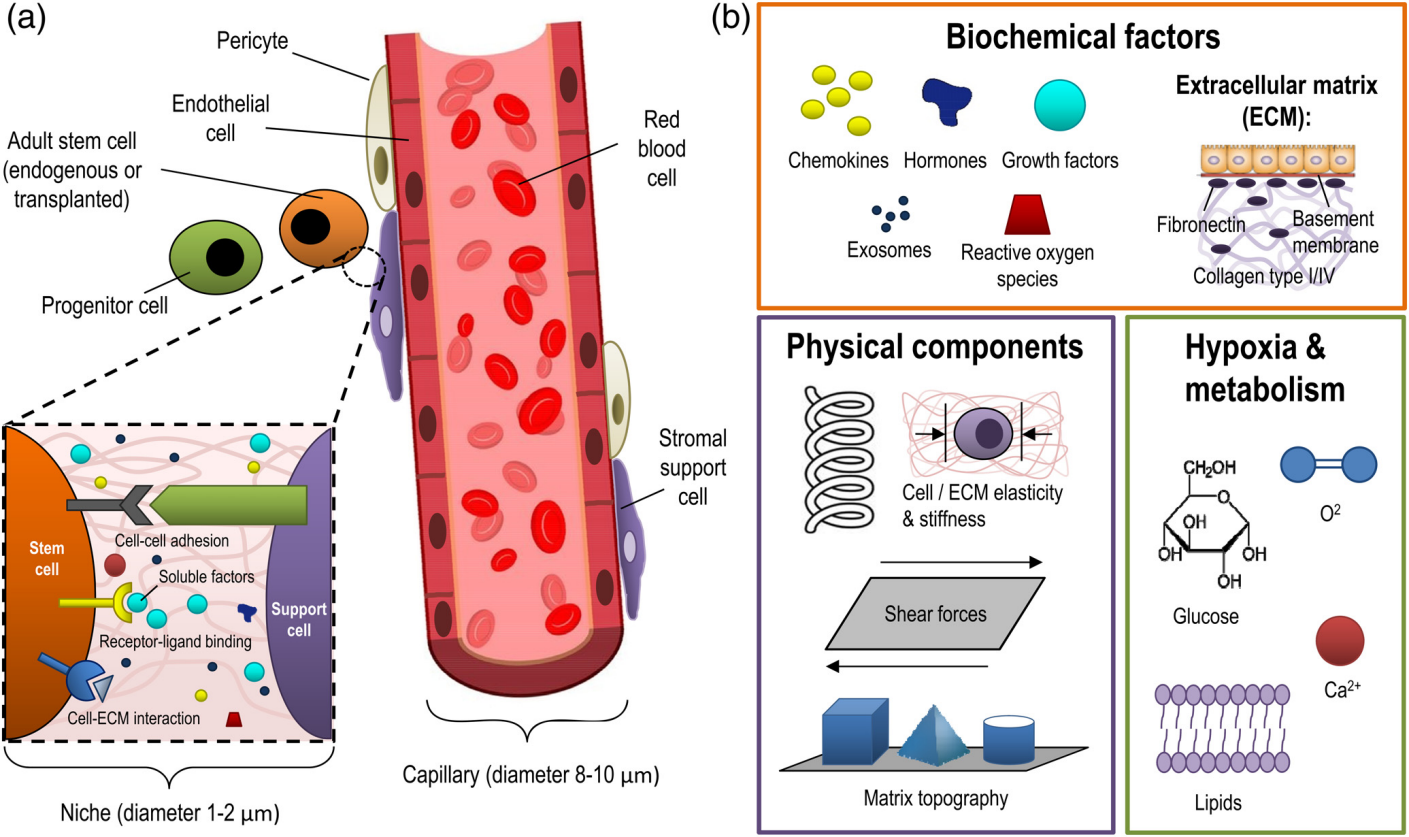
The adult stem cell niche. (a) Anatomy of the niche. The ASC niche is a dynamic, *in vivo*, microscopic microenvironment associated with a perivascular location, and/or have supporting cells which contribute cell‐cell, cell‐extracellular matrix (ECM), or cell‐soluble factors to the niche to maintain ASC in a quiescent state. They are believed to be on the order of 1‐2 μm in size within the area of the ASC. Changes in state present extrinsic changes which can give the ASC information needed regarding whether to divide and participate in homeostasis versus tissue repair. (b) Components of the niche. The niche includes: 1) soluble biochemical cues and ECM components 2) poorly understood physical signals, including possible roles for elasticity, stiffness, shear forces, and aspects of ECM such as topography 3) metabolic aspects such as oxygen, glucose, and calcium

ASC accomplish these divisions not only because of specialized molecular machinery but also because of specialized external microenvironments, termed niches, which support function of ASC (Figure 2). Fundamentally, niches must protect ASC from loss, because if all ASC were lost, then the tissue and organism would not survive. Not surprisingly, niches are complex multidimensional environments that change in space and time, are located throughout an adult tissue where “ASC” are present, possess unique anatomical and functional dimensions, and have been reviewed in detail.^35^ They were first experimentally identified in fruit fly (*Drosophilla melanogaster*) in solid tissue within the developing ovary models and have been studied in the hematopoietic and the hematopoietic and skin models and other model systems.^36^ In the fruit fly ovary, this stem cell niche is maintained, in part, by intercellular interactions between germ stem cells and the somatic cap cells,^37^ and signaling factors like decapentaplegic (Dpp), which are bone morphogenetic 2/4 protein (BMP 2/4) analogues. These niches are on the order of 5 μm × 0.5 μm × 2 μm and tend to contain niche cells with specialized functions, including unique expression of cell surface receptors, soluble extracellular matrix for supporting stem cells and the microenvironment for maintaining the state of ASC (Figure 2). These highly specialized stem cell niches serve as a controlled microenvironment that, when altered due to physiological and pathological stress, control how ASC respond.

A major question is, how are functions of ASC evaluated? The development of functional assays has been critical for identifying unique markers for stem/progenitor populations. These assays have provided a framework for purifying stem cells and for understanding quantitative differences in the cell's *in vivo* differentiation properties.^38^ To define the true properties of ASC, like self‐renewal, stem cell hierarchy, and stem cell niches, *in vivo* assays are critical. Typically, this involves isolation of ASC from mouse or human donor tissue, clearing of endogenous tissue in host organ, which contain ASC within their niches, and orthotopic transplantation of donor ASC in the host organ.^38^ The donor ASC self‐renew, differentiate, and their functions can be assessed, typically by removing the host tissue and analyzing for tissue growth, differentiation, and self‐renewal. A key assay for assessing stem cell fates is lineage tracing,^39^ which is a method for understanding the descendants of an originating cell that is marked. Using vital dyes, radioactive labels, genetically encoded reporters, or conditional Cre‐Lox technology, stem cell scientists can track the fates of cells, including the number of cell divisions (low vs. high), their location, and the relative time in which they arose.

Differentiation of ASC occurs either spontaneously or due to a change in microenvironment. Stem cells leave their niche and differentiate into one or more progenitors, which then are committed to multiple lineages. From a molecular point of view, differentiation essentially suggests a change in cell state. The cell state is defined by cell‐specific transcription factors (TF), which activate cell‐specific genes at the DNA level, which result in the production of cell‐specific proteins, which in turn confer cell identity and function.^40^, ^41^ The TF that control cell states are often controlled by developmental enhancers which contain binding sites for TFs from earlier states. Furthermore, expression of these TFs and access to these developmental enhancers are controlled epigenetically.^42^ The presence of quiescent, primed, and active promoter/enhancers thus classifies each gene themselves in multiple states, with progenitor cells having primed states and more mature cells having active or open enhancers at differentiation genes. To establish a particular state, developmental or lineage‐specific TF also often will have to repress opposing states (endothelial vs. cardiac differentiation of a cardiovascular progenitor cell), or previously committed states (activation of liver specific TF repressing previous states in endoderm progenitor cells). The former would guarantee that cells form the correct fate and repress alternate fates, whereas the latter enables cells to move “forward” with differentiation without dedifferentiating in the backwards direction. Ultimately, genes associated with mature differentiation are diverse, ranging from cell surface markers or receptors, secreted proteins, or cell‐specific enzymes.

Scientists who support the cancer stem cells (CSC) hypothesis also believe that CSC (or tumorigenic stem cells), similar to ASC, sit at the top of a hierarchy, in which the cells within the hierarchy represent the tumor.^43^ Therefore, cancer can be viewed through the eyes of regenerative biology. CSC theory proposes that the tumor hierarchy is a “caricature” of normal cellular hierarchy, with a CSC at the top of the hierarchy. Experimental evidence suggests that CSC are a rare population of cells within a tumor, which can undergo self‐renewal and can give rise to the entire tumor, including recreation of the parent tumor's histology. This is consistent with the appearance of the differentiated state of tumors in biopsies. CSC theory explains tumor heterogeneity, or the fact that tumors are believed to be clonal, even though the tumor cells themselves are heterogeneous and nonidentical. CSC share the property of self‐renewal with ASC.^44^ CSC theory predicts that only a small number of cells of the tumor can in fact give rise to the tumor, whereas the remaining cells are more differentiated and are destined to die and proliferate less. CSC may arise from normal stem cells that have oncogenic mutations, as they are much longer‐lived than their differentiated progeny.^45^ CSC also may arise from tissue progenitors that have gained oncogenic mutations which enable the ability to self‐renew, which can lead to tumor formation.

### 2.3 The advent of and applications of PSCs

Although ASC represent evolution's approach for growing and maintaining the tissues in the body, PSC, including both ESC and induced iPSC, together represent a biotechnology with numerous health applications. The value of self‐renewing PSC is that in theory, an infinite number of therapeutic cells can be generated from a single clone, and that these cells are personalized, that is, generated from an individual person and genome.

Through disparate studies of the regeneration of organisms like planarians, human tumors like teratomas and germ cell tumors, and experimental studies of the zygote (fertilized egg), scientists theorized and developed the concept of pluripotency. Supporting this notion, they found that portions (inner cell mass) of the developing zygote, or the pre‐implantation blastocyst, can be cultivated to form a cell that meets the stringent criteria of pluripotency.^7^, ^8^ These ESC self‐renew, differentiate *in vitro*, and could be introduced into the embryo to give rise to chimeric mice in which components of all three lineages are donor derived. Techniques have been developed such that the donor cells could be genetically modified using transgene or knock‐in technologies.^46^ Thus, the recipient mice can have donor cells, which are genetically modified for a particular disease phenotype, which can be passed through the germline to create new transgenic mice. When transplanted subcutaneously in immunodeficient mice, these cells give rise to teratomas, which are tumors derived from all three germ layers. Techniques to grow the mouse‐derived ESC (mESC) *in vitro* enabled understanding of self‐renewal, differentiation toward germ layers, followed by specification and maturation using lineage‐specific protocols based upon mouse development.^47^, ^48^, ^49^ Scientists found that developmental gene networks function in mESC similarly to how they function in lower organisms.^40^ Studies of the maturation of mESC and development of reversal of disease in mouse models of organ failure commenced next.^50^, ^51^, ^52^ The development of human ESC was a huge step forward and used similar techniques and relied on similar differentiation approaches. Furthermore, the development and commercialization of cultivation techniques for hESC^53^, ^54^ and the differentiation and transplantation of hESC have helped to push ESC biology forward and demonstrated their potential in academic labs for both potential therapeutic applications^55^ and clinical studies in patients.^56^, ^57^

Despite the promise of hESC, the ethical issues of handling discarded human fetuses generated tension in the field. In this environment, the advent of genetic technology to reprogram any mouse or human adult, somatic, cell into a human‐iPSC was a huge discovery and a great boom to the field.^58^, ^59^ iPSC could be generated by simple, exogenous expression of four transcripts, or factors, Klf, Sox2, Oct 4, and c‐Myc, in contrast to other techniques like nuclear reprogramming and cell fusion, which are far more complex and inefficient.^60^ This combination of TF remodels chromatin to enable concurrent gene activation and repression to result in activation of endogenous pluripotency factors and a pluripotent state highly similar to ESC. This personalized approach enables rapid generation of personalized cell lines, particularly from patients with genetic diseases, or as donor cells that can be differentiated for transplantation. Importantly, iPSC bypass the ethical issues related to ESCs and ideally, the immune barrier of transplanting allogeneic cells for therapy. For example, a recent promising study demonstrated the use of iPSC for retinal transplantation and resulted in the first transplanted iPSC‐derived cells in patients.^56^ Despite this landmark, the results were halted due to mutations and heterogeneity in clones that occur *in vitro* during the reprogramming process. Challenges to PSC implementation include the genetic fitness of the cells (lack of mutations or chromosomal aberrations), the prevention of immune rejection, the avoidance of cancer, measuring functional maturity of cells, scale up,^61^ and understanding *in vivo* cell fate. iPSCs have many other applications for *in vitro* disease modeling and drug development not mentioned here.

### 2.4 Cell‐based therapies are more complex than other established therapies

The clinical application of cell therapy, often using stem cell‐derived products, has reached center stage. In the last 15 years, both ASC and PSC‐derived cells have proceeded through preclinical models, have paved the way for commercialization, and have motivated numerous clinical trials.^57^ As patients continue to die waiting for a donor organ on organ transplantation lists, stem cell and tissue engineering‐based approaches offer hope. Examples of cell therapies include chimeric antigen receptor (CAR‐T) cells for cell‐based cancer immunotherapy,^62^ cardiac cell therapy,^63^ islet cell therapy for type II diabetes mellitus,^61^ retinal progenitor cell therapy for macular degeneration,^56^ hepatocyte cell therapy,^64^ cell therapy for the nervous system,^65^ and mesenchymal stem cell (MSC) therapy for a wide range of diseases.^66^ Consistent with this, forecasts suggest that the number of studies using adult and PSC will continue to grow because of the increasing need for treatments for chronic diseases.

**Figure 3:**
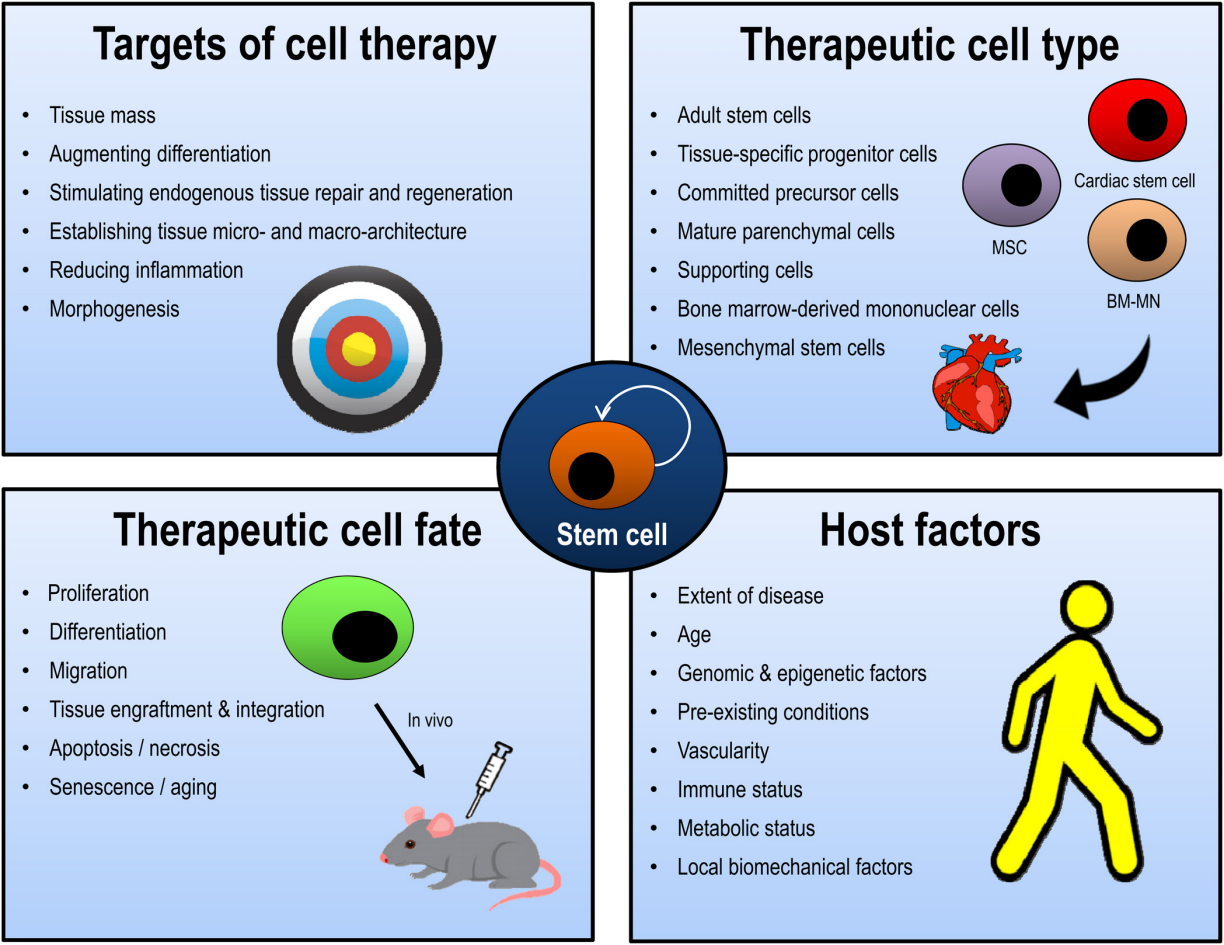
Hurdles for engineering cell and tissue therapies. Cell therapies, often using ASC, progenitor cells, or human pluripotent stem cell‐derived products, will face numerous hurdles compared to therapies like pharmaceuticals, biopharmaceuticals (monoclonal antibodies), medical devices, or even surgical treatment. Targets of cell therapy: most therapies have a highly specific target. However, cell therapies are complex, and their targets are numerous. They include increasing tissue mass, differentiation (with all its complex stages), morphogenesis, stimulating endogenous tissue repair and regeneration, establishing tissue micro‐and macro‐architecture, and reducing inflammation. Therapeutic cell fate: the cells being transplanted may have many fates which complicate cell therapy, because their fate may vary between patients. For example, proliferation, differentiation, migration, tissue engraftment and integration, apoptosis/necrosis, and senescence/aging. Therapeutic cell type: many cell types are available for therapeutic cell replacement, and it is unclear which therapeutic cells are the best for a particular case. Cardiac cell therapy is one example in which this is the case. For example, possible cell types include adult stem cell, tissue specific‐progenitor cell, committed precursor cell, mature parenchymal cell, supporting cells, bone marrow‐derived mononuclear cells, and mesenchymal stem cells. Host factors: Unlike other types of therapies, host factors play a major role in dictating the fate of cell therapy. However, many of these effects are unknown. Potential factors include extent of disease, age, genomic and epigenetic (intrinsic) factors, pre‐existing local or systemic conditions, vascularity, immune status, metabolic status, and local biomechanical factors. Currently, these can only can be determined empirically, and many animal models do not take these factors into account in preclinical models

To imagine the molecular imaging of cell therapies, it is important to distinguish cell therapies from traditional therapies like medicine and surgery in many nonobvious ways, (Table 1, Figure 3). For example, surgical therapy, for localized congenital or acquired disease, can be monitored visually within the operating room and has predictable complications such as bleeding, infection, and pain. Knowledge of these side effects is based upon knowledge of the coagulation system, immune system, and nervous system, respectively. The same can be said of many medical devices associated with surgical problems that are used in therapy (i.e., intra‐aortic balloon pump). Pharmaceuticals or biopharmaceuticals have many aspects that are predictable. A small‐molecule drug, or even a therapeutic monoclonal antibody, having gone through $1 billion drug development process, has a known molecular target, a highly specific receptor or enzymatic target within the cell, with predetermined therapeutic doses and side effects. The absorption, distribution, metabolism, and excretion (ADME) of the drug occur by known, somewhat predictable pathways. Similarly, the routes of administration are well known (i.e., intravenous, oral) and result in predictable changes in ADME. In that sense, surgery, medical devices, and pharmaceuticals/biopharmaceuticals are quite predictable.

**Table 1:** Targets of biotherapies

Therapy name	Target	Classification
Linisopril (Prinivil, Zestril)	Angiotensin converting enzyme	Pharmaceutical
Humira (adalimumab)	Tumor necrosis factor alpha inhibitor (TNFα)	Biopharmaceutical (monoclonal antibody)
Intra aortic balloon pump	Increased myocardial oxygenation	Medical device
Transplantation	Organ replacement (kidney, liver)	Surgery
Regenerative medicine	Restoring tissue function, increased tissue mass, differentiation, morphogenesis, establishing tissue architecture, stimulating endogenous repair, supporting endogenous tissue regeneration, and reducing inflammation	Cell/tissue therapy

Cell and tissue‐based therapy contrasts starkly with both traditional surgical (or medical device based) and pharmaceutical‐based therapies (Table 1, Figure 3). The cells themselves are alive, complex, and capable of multiple fates based in cell biology, such as senescence, aging, proliferation, differentiation, migration, apoptosis/necrosis, tissue engraftment, and integration. The pretransplant stages, including cell isolating techniques, cultivation conditions, medium, passage rate, and recovery methods not only may vary between studies but also may influence results. Similarly, the targets of cell therapy are widespread. For example, increasing tissue mass, augmenting differentiation, morphogenesis, establishing tissue micro‐ and macro‐architecture, stimulating endogenous repair and tissue regeneration (i.e., angiogenesis), and/or reducing inflammation are often all objectives of cell therapy. Host factors, such as extent of disease, age, genomic and epigenetic factors, pre‐existing conditions, tissue factors (vascularity), immune status, metabolic status, and local biomechanical factors also likely play a role in dictating the success of cellular therapy. The cells themselves can be of various types (bone marrow‐derived mononuclear, MSC, ASC, adult committed progenitor cell, mature parenchymal cells, supporting cells) and therefore it is often unclear if they should be used alone or in combination with other cells, as they are in a tissue‐based construct. Overall, the obvious complexity in cell fate, targets, host factors, and cell sources (Figure 3) raises several questions regarding cell therapy and suggests that new tools are needed to evaluate cell therapies in living systems. This underlying complexity is advantageous for chemical and biological engineers to investigate, which aligns with their training in complex systems. Molecular imaging may provide major solutions to this problem by allowing researchers to evaluate delivery, predict *in vivo* biology, and assess efficacy in preclinical models and individual patients.

## 3 TARGETS OF MOLECULAR IMAGING IN REGENERATIVE MEDICINE

### 3.1 Overview of molecular imaging

The purpose of our detailed of review of regenerative medicine and stem cells is to elucidate numerous opportunities for molecular imaging, which are discussed in detail below. Molecular imaging is a branch of radiology and imaging, which focuses on quantitative imaging noninvasive molecular events in living subjects,^67^ similar to the concept of the noninvasive biopsy. The focus is on high sensitivity imaging in living subjects because the numerous biological pathways that have been elicited are 100% intact, and numerous cell types within a tissue are at the correct location and proportion. Further, the main objective is to image the biology of the process, rather than the anatomy (location of an organ) or function (blood flow). The advantage of imaging is that each living subject, in a preclinical (i.e., mouse) or clinical study, can serve as its own control. High sensitivity together with methods to generate and quantitate an imaging signal are needed in this field, which can allow detection of molecular events that occur either with a low mass of substance of interest, or at low concentrations.

A simple example is illustrative of molecular imaging. If a vascular endothelial growth factor receptor (VEGFR) inhibitor is being administered for cancer, then the therapist would need to assess the extent of the target VEGFR expression within the cancer prior to therapy. Thus, a molecular image would first be generated prior to therapy. It would guide the decision to administer the molecular therapeutic, which in this case is the VEGFR inhibitor. Post‐therapy, it would be important to assess whether the VEGFR inhibitor was targeted, which presumably resulted in loss of tumor and loss of target. Therefore, another molecular image would be generated, which could be used to assess the previous therapy, including factors such as dose and efficacy. Not only molecular imaging is tied to therapy and drug development, but it is also connected with early disease detection. It is believed that molecular changes must occur prior to anatomical changes and thus molecular imaging and thus molecular imaging can be used for early detection of disease.

Keeping this overview in mind, below we will identify potential targets of molecular imaging in regenerative medicine. In the subsequent section, we will separately review the state of cell imaging as it pertains to regenerative medicine.

### 3.2 Molecular imaging targets in tissue engineering

The molecular targets for imaging in tissue engineering are dependent upon which cell type is being tissue engineered (i.e. epithelial, neural, connective, muscle). Molecular targets include gene expression of individual extracellular matrix proteins, individual protein concentrations, composition of extracellualr matrix, and potentially, extracellular matrix strength (biomechanics). Molecular imaging of the location or degradation of a biomaterial/scaffold of interest. Molecular imaging of biological pathways and tissue microenvironment would be critical to evaluate the relative success/failure of tissue engineered constructs in unique environments, such as acute versus chronic inflamed tissues. Conventional anatomical imaging and functional imaging can be used with molecular imaging in unique ways, for example, imaging blood vessels, flow, and angiogenesis receptors.

### 3.3 Molecular imaging targets in adult (cancer) stem cells and regenerative biology

The emerging details in regenerative biology, mentioned earlier, suggest that numerous molecular imaging targets exist for tissues *in vivo*, and that noninvasive molecular imaging and analysis of these targets could deepen our knowledge, but also hasten new diagnostics and therapeutics development. Molecular imaging the biology of ASC, such as cell receptors or self‐renewal pathways, is particularly challenging, because of the scarcity (< 1%) and size of these cells. However, obtaining *in vivo* information about the biological aspects of multipotent progenitors would also be valuable. Multiplex (more than 1) imaging of key molecular targets of the cellular hierarchy present within tissues could provide valuable noninvasive assessment of tissues, which is currently not possible. This may include imaging of molecular interactions between key cell populations within tissues, and how they change with time and space. Molecular imaging of the stem cell niche has not been established, and not only the location of the niche but also the molecular composition of the niche, could be valuable for understanding tissue states. The simple integration of noninvasive molecular imaging with ASC assays, which are typically endpoint, can provide new information to stem cell scientists, and we have previously pursued this approach.^38^ Noninvasive molecular imaging of ASC differentiation, one of the main purposes of ASC transplantation, would be valuable for understanding *in vivo* cell fate during various types of tissue insults. However, as ASC typically differentiate into more than cell type, targeting differentiation would involve imaging multiple cell types. This may involve with cell differentiation‐specific transcription factors or lineage specific proteins, or other potential molecular targets. *In vivo* ASC differentiation is accompanied by morphogenesis, interactions with the tissue environment, and tissue remodeling, all of which represent molecular targets. Because of isolation and characterization of CSC, scientists interested in regenerative medicine and molecular imaging can apply similar principles to imaging CSC, including the CSC niche, differentiation, morphogenesis, and tissue remodeling. In summary, studying ASC biology in the living subject using molecular imaging has enormous potential.

### 3.4 Molecular imaging targets in pluripotent stem cell‐based therapy

As mentioned earlier, PSCs represent a powerful technology. Unlike ASC, the *in vivo* applications of PSC typically first involve cell transplantation prior to imaging. The main applications of *in vivo* molecular imaging are similar to those of ASC, including niche, differentiation, morphogenesis, tissue microenvironment, and tissue remodeling. These are specific for each cell and tissue type. Other applications could involve imaging tissue integration, tissue function, the immune response, and tumorigenicity.

### 3.5 Molecular imaging targets in cell therapy

Molecular imaging of cell therapies begins with cell imaging, which will be discussed further below. Furthermore, many of the applications for ASC and PSC also hold for cell therapies in general. Future directions for these cell therapies are discussed in the “Next generation regenerative medicine” section.

## 4 MOLECULAR IMAGING‐BASED CELL IMAGING FOR REGENERATIVE MEDICINE

### 4.1 Key factors for imaging and quantifying cells

Molecular imaging‐based cell imaging is a major technique that has been in development for over 20 years. A major question is, how many cells can be detected in a particular location, or what is the sensitivity for cell imaging? Detecting fewer and fewer cells, thereby approaching single‐cell imaging, is one of the goals of the field, and could be applicable in preclinical or clinical models. The sensitivity for cell imaging is a function of several factors. Cell imaging can only be accomplished either by engineering an imaging signal within or upon (i.e., cell surface) cells of interest using reporter genes, molecular probes, both, or by taking advantage of intrinsic mechanisms that generate imaging signal/contrast compared to neighboring cells. Normally, signal is proportional to the mass of the cell population, assuming each cell has a similar mass of contrast/probe. However, target cell signal must be normalized or subtracted from background cell signal. Sensitivity is normally measured in units of molarity and represents the concentration of imaging probe or agent, with a range reported from 10^−4^ to 10^−18^ M, and for cell imaging sensitivity can also be measured number of cells detected. Sensitivity measurements are affected by whether the imaging instrumentation is whole body, whole organ, endoscopic and/or microscopic. Furthermore, instruments that use a focused beam of excitation (microscopic) and/or are closer to the tissue (endoscopic) potentially have higher sensitivity. Other critical imaging parameters include spatial resolution, which varies between submillimeter to ∼8 mm for imaging instruments, depth resolution, which varies from 100 μm to cm of tissue, temporal resolution/time of acquisition, which varies from 1 ms to 15 min, and field of view, which varies between mm of tissue to the whole body.

### 4.2 The value of cell imaging

Key questions in regenerative medicine can be answered with cell imaging (Figure 3). Cell imaging assists in evaluating location and magnitude of therapy and can be used to optimize cell delivery and cell dose. It can be used to identify the fate of stem cells after transplantation, including cell engraftment, cell viability and death, cell integration, cell proliferation, and cell differentiation. Cell imaging can help to determine the efficacy of cell therapies by comparison of therapeutic cell types. Cell imaging can be used to evaluate cell delivery techniques, (direct injection vs. intravenous injection) and its effect on efficacy. Finally, cell imaging can be used to impact host factors (age, ethnicity, gender, immune system, etc.) on cell fate (Figure 3). Unfortunately, very few clinical trials use cell imaging techniques,^68^, ^69^, ^70^ so these approaches have yet to formally reach the clinic.

## 5 CELLULAR PRELABELING AND CELL IMAGING WITH NANOPARTICLES

### 5.1 History

Noninvasive cell imaging using cell prelabeling is a well‐established technique, which enables greatly improved sensitivity upon cellular uptake, as mass (mg) doses of imaging agent can be delivered intracellularly prior to imaging. Two early technological developments, the development of 111‐indium oxine radiotracer^71^ and of superparamagnetic iron oxide (SPIO) nanoparticles (NP)^72^ were critical. These techniques enabled prelabeling of cells prior to therapeutic cell injection in small animals and patients. 111‐Indium, within the 111‐indium oxine, is chelated by subcellular components by exchange and release of the oxine, the 8‐hydroxyquinoline carrier.^73^ After 111‐indium oxine labeling, cells can be imaged with single‐photon emission computed tomography (SPECT), which has the ability to detect gamma photons. Following SPIO NP labeling using standard transfection reagents,^74^ cells can be imaged with MRI. In the subsequent section, we will focus on experiments in which cells are prelabeled with NP prior to *in vivo* injection.

### 5.2 NP uptake *in vivo*

NP are generally sized from 1 to 100 nm and composed of metals, semiconductors, or polymers and exhibit unique properties, which can be advantageous for *in vivo* imaging. In this review, we are focused on cell prelabeling. However, many studies employing NP use NP injected alone, as opposed to prelabeling, and intravenously inject NP in the living subject (mouse), followed by histochemical analysis demonstrating cellular uptake, often within tumors. In addition to tumor uptake, phagocytic cells, like circulating monocytes, tissue macrophages (Kupfer cells in the liver), dendritic cells, and neutrophils can be labeled by NP injection *in vivo*. Importantly, these phagocytic cells are more readily prelabeled *in vitro* than ASC, hPSC‐derived progenitors, or mature hPSC‐derived cells of ectodermal, endodermal, or mesodermal origin. These latter cells require optimization of *in vitro* transfection and have not been routinely challenged *in vivo*.

### 5.3 MRI cell imaging with NP

**Figure 4:**
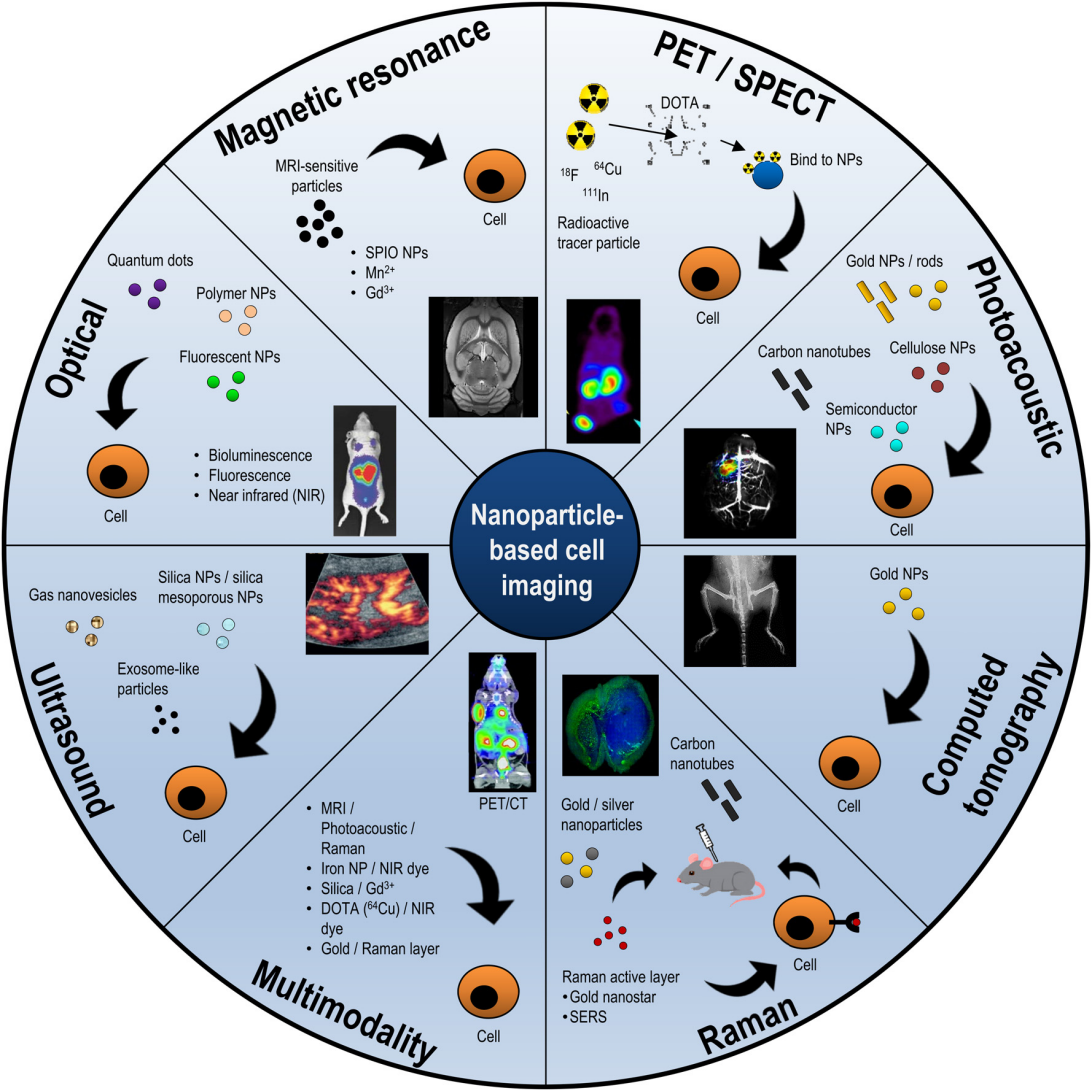
Nanoparticle‐based imaging strategies. In recent years, a wide range of NP have been synthesized and characterized. Many of these NP either have intrinsically or extrinsically engineered capability for *in vivo* imaging. In this figure, we summarize the wide range of imaging modalities that have been used for NP‐based cell imaging. In nearly all cases, NP are prelabeled into cells, cells are injected in a living subject, and cells are imaged by the technique listed. In some cases, NP alone are directly injected in the living subject but accumulate at the cells of interest

The advent of cell prelabeling with SPIO NP established an interdisciplinary field between regenerative medicine and materials science (nanotechnology), chemistry (solid state, surface), physics (magnetism, nuclear, optical), radiology/imaging (MRI, SPECT), chemical and biological engineering (particle synthesis, cell targeting, binding, intracellular delivery), pharmacokinetics, and toxicology. How NP can be used therapeutically within regenerative medicine^75^ and how nanomaterials can dictate stem cell fate has been recently reviewed.^76^ SPIO NP are ferrous oxide crystals which exhibit paramagnetic properties and have been widely used in radiology as contrast generating agents and for cell imaging. Proper coating of the particles, with molecules like albumin, sugars (dextran), and hydrophilic polymers, enable cellular uptake from a “ferrofluid.” After transfection, about 10‐100 pg of iron/cell has been measured. Cellular toxicity has been shown to be minimal, although it is unclear how different cell types handle these particles. Furthermore, how the particles are distributed in a subcellular fashion has not been clearly delineated. SPIO NP create imaging contrast locally by shortening T2 relaxation time, which results in a loss of signal on T2, and darker image on conventional, or T2* weighted MRI‐sequences. Both the absolute voxel size of the signal loss and the intensity of signal loss are proportional to the number of particles/labeled cells.^77^, ^78^ This approach is called molecular MRI, because it involves enhancing sensitivity of MRI for molecular imaging (and cell imaging). Typically, MRI has an approximate sensitivity of 10^−4^ M, which is much lower compared to approximate sensitivities for positron emission tomography (PET) (10^−12^ M). Despite its lack of sensitivity, MRI offers many levels of signal modulation for improved cell imaging, including design of specialized pulse sequences to vary magnitude of radiofrequency (RF) pulses, engineering of specialized coils to receive RF information with higher sensitivity, ability to vary acquisition times, and increased signal strength with newer MRI machines that have stronger magnets. Studies have determined sensitivity by prelabeling cells with SPIO NP, injecting into mice, and performing MRI. In small animals, the sensitivity reported, in terms of cell number, is approximately 3 × 10^5^ to 1 × 10^6^ for dendritic cells^79^ with a 1.5 MRI scanner. Furthermore, single cell MRI in mouse breast has been reported^80^ and validated by a second technique, which demonstrates the ability to optimize multiple MRI parameters, including extended acquisition times and specialized engineered coils. Using a clinical 1.5 T MRI scanner, a swine study with MSC injected in the heart demonstrated ∼1 × 10^6^ SPIO‐labeled MSC,^81^ and using a 3 T MRI scanner, we recently reported at least 1.51 × 10^7^ MSC SPIO‐labeled in the swine heart.^82^ Positive contrast MRI imaging approaches have been developed, and a recent study, employing manganese NP, reported serial *in vivo* imaging of 2.1 × 10^6^ MSC in the hips of rats, using 3.0 T MRI scanner.^83^ This information is summarized in Figure 4 and Table 2.

**Table 2:** Cell imaging modalities and key features

Modality	Temporal resolution	Spatial resolution	Penetration depth	Sensitivity	Cell sensitivity, reporter gene imaging (*in vivo*)	Cell sensitivity, nanoimaging (*in vivo*)
CT	Minutes	50‐200 μm (preclinical), 0.5‐1 mm (clinical)	Limitless	Undetermined	Not applicable	~10^5^ cells
IVM	Seconds to days	1‐10 μm	~700 μm	10^−15^ to 10^−17^ M	Single cell	Single cell
MRI	Minutes to hours	25‐100 μm (pre‐clinical), ~1 mm (clinical)	Limitless	10^−3^ to 10^−5^ M	~10^7^ cells	~10^5^–10^6^ cells
Optical (BLI)	Seconds to minutes	3‐5 mm	1–2 cm	10^−15^ to 10^−17^ M	Single cell	unknown
Optical (Flourescence)	Seconds to minutes	2‐3 mm	<1 cm	10^−9^ to 10^−12^ M	Single cell (gfp application)	~10^5^ cells (quantum dot application)
PA	Seconds to minutes	10 μm‐1 mm	6 mm‐5 cm	Undetermined	~10^6^ cells	~10^5^ cells
PET	Secondsto minutes	1‐2 mm (preclinical), 5‐7 (clinical)	Limitless	10^−11^ to 10^−12^ M	~2 × 10^8^ cells	~10^6^ cells
SPECT	Minutes	1‐2 mm (preclinical), 8‐10 mm (clinical)	Limitless	10^−10^ to 10^−11^ M	~1 × 10^8^ cells	~10^6^ cells
Raman microscopy	Minutes to days	~1 mm	~5 mm	10^−12^ to 10^−15^ M	Not applicable	~10^5^ cells
US	Seconds to minutes	~1‐2 mm for deep‐tissue applications (few cm depth)	~1 mm‐1 cm	10^−12^ M (microbubble application)	Not applicable	~10^4^–10^5^ cells (microbubble application)

CT = computed tomography; IVM = intravital microscopy, MRI = magnetic resonance imaging; BLI = bioluminescence; PA = photoacoustic; PET = positron emission tomography; SPECT = single‐photon emission tomography; US = ultrasound.

### 5.4 Optical and near‐infrared cell imaging with NP

Optical imaging is typically the first approach used for cell prelabeling and *in vivo* imaging. Optical *in vivo* imaging is affected by factors that influence light interaction and transport within tissues, including photon back reflection, refraction, diffusion, absorption, and scattering. These wide range of fates, and the many mechanisms available to modulate light source and light path, are the reason a wide range of *in vivo* optical imaging techniques are available. These techniques include whole body (small animal) optical imaging instrumentation,^84^ with the common commercial instruments being. The Maestro™ (PerkinElmer, Waltham MA), Clairvivo OPT (SHIMADZU, Kyoto, Japan), and IVIS Imaging System (PerkinElmer, Waltham MA).^85^ Other optical techniques include diffuse optical tomography,^86^ fiber optic‐based microendoscopy,^87^ intravital and multiphoton microscopy,^38^ optical coherence tomography,^88^ raman microscopy,^89^ photoacoustic imaging,^90^ and biophotonic sensing.^91^

For optical imaging, NP (and conjugated fluorophores) are excited and can emit in both the near infrared (NIR) window (650‐900 nm),^92^ and the NIR II window (1,100‐1,400 nm).^93^ These optical windows are highly valuable for *in vivo* imaging. Advantages include cost, ease of preparation, multiplex (detecting multiple events), and cost and availability of instrumentation. During NIR imaging between the optical absorption windows of H_2_O and hemoglobin, light penetration occurs more readily and deeper into tissues, as dictated by the Lambert–Beer law.^94^ Based on this law, for example, blue photons (450 nm) have a mean absorption length of about 0.4 cm, whereas NIR photons (800 nm) have a length of 1.8 cm.^94^ Smart multimodal probes, employing functional NIR fluorophores conjugated to SPIO NP, were developed as early as 2002.^95^, ^96^ Ideally, these agents either can be functionalized onto the surface of commercially available nanospheres (latex or polystyrene)^97^ or a NP of interest, or can be fully incorporated within a polymeric NP.^98^ Polymer NIR NP, or P dots, may suffer from self‐quenching due to molecule aggregation, but there are promising solutions.^98^ These NIR dye‐containing NP have a typical core‐shell structure in which the core is an NIR organic dye and the shell is a polymer or inorganic matrix‐based particle.^99^ Regardless of which NIR NP are used for cell imaging, there are many key parameters for obtaining an accurate, reproducible, and clear image. These include a high molar absorption coefficient at the excitation wavelength (absorbance divided by the product of path length and concentration) and quantum yield (number of emitted photons per absorbed photons), the product of which is brightness. The brightness enables visualization of labeled cells over background in NIR applications. Along these lines, commercially available fluorescent and NIR NP have prevented unwanted NIR dye leaching, quenching, and photobleaching by incorporating dyes into their polymer matrix^97^ and several types of fluorescent and NIR‐emitting particles are available.

Quantum dots (QD), another class of NP, are nanocrystal, semiconductor clusters with unique electro‐optical properties. Within these QD, at sizes smaller than the Bohr exciton radius (a few nanometers), energy levels are quantized. QD exhibit tunable fluorescence and NIR emission with a change in diameter, as this influences quantum confinement energies of electron‐hole pairs.^4^, ^100^, ^101^ The modification of synthesis techniques has enabled uniform production and application of QD in biological systems. For cell prelabeling and optical cell imaging, QD hold several advantages, and concerns about metal toxicity have been alleviated through surface modification.^85^ Compared to conventional fluorophores, they enable both a broad absorption and an extremely narrow emission spectra, accompanying enhanced Stokes shift. Furthermore, they demonstrate a high quantum yield, long fluorescent lifetime, enhanced photostability, and reduced photobleaching.^85^ Although many factors affect sensitivity of cell imaging *in vivo*, subcutaneous implantation demonstrates detection of approximately ∼5 × 10^4^ stem cells labeled with QD655, which emits at 655 nm.^102^ Furthermore, improved deep tissue (lung, liver) imaging together with biodistribution studies have been demonstrated with adipocyte stem cells labeled with QD800 in emphysema and liver failure models.^103^ Consistent with this, using QD800, 1 × 10^5^ cells, but not 1 × 10^4^ cells, could be detected in subcutaneous transplantation experiments with ESC.^104^ This information is summarized in Figure 4 and Table 2 **.**

### 5.5 Raman cell imaging with NP

The intersection of Raman imaging with regenerative medicine has recently been reviewed in detail.^105^ Raman is based on the principle by which light is not only elastically scattered (Rayleigh scattering, in which photons contain the same energy, frequency, and thus wavelength of incident light) but also inelastically scattered, in which photons can have less energy and frequency, termed Stokes scattering, or more frequency, termed anti‐Stokes scattering. This Raman effect, for which CV Raman won the Nobel Prize in 1930, is a function of the natural changes in vibrations and stretching motions of chemical bonds within the sample. The Raman shift between incident and inelastically scattered light can be detected at different wavelengths using Raman spectroscopic techniques and represents a unique signature for each molecule, and a particular molar mixture of multiple molecules. However, typically only ∼1 in 10^7^ photons demonstrate the Raman effect. Raman molecular imaging is advantageous because of its high specificity and multiplex capability and is a widely used analytical technique. A disadvantage of Raman is depth penetration, and increased temporal resolution required for detection. Despite this low sensitivity, label‐free cell imaging *in vitro* has been accomplished and is typically used to distinguish lipids, proteins, and DNA (phosphate) and can distinguish stem cell types during differentiation *in vitro*.^106^ Raman NP imaging is based upon surface enhancing Raman spectroscopy (SERS) effect, which greatly enhances Raman sensitivity. Here, an enhanced (up to 10^14^ increase) in Raman signal (shift) occurs if a molecule is adsorbed at the interface of a noble (Au or Ag) metal which is curved and/or roughened. This SERS enhancement enables high sensitive imaging. This works because the conduction band of the metal within the NP can generate surface plasmon resonances at the surface due to collective oscillation of electrons, or can participate in electron transfer from the Raman‐sensitive material.^107^ If multiple particles are each engineered with different Raman‐sensitive molecules, multiplex imaging *in vivo* is possible,^108^ in which multiple flavors of molecules can be imaged. Cell prelabeling studies have demonstrated that 5 × 10^5^ HeLa cells, which overexpress folate receptor, can be visualized *in vivo* in the mouse ear using Raman imaging when prelabeled with folate‐conjugated SERS particles.^109^ Consistent with this, the sensitivity of Raman NP imaging with conventional SERS AuNP has been shown to be detectable at picomolar levels *in vitro* 89 and 5.5 mm depth resolution was achieved with low nanomolar concentrations of sensitivity.^89^ Engineering SERS AuNP with new “star‐like” shapes to enhance resonance effects, with resonance in the NIR region and in tune with the 785 nm laser, enabled significantly improved sensitivity (femtomolar),^110^ and improved Raman dyes adsorbed on these AuNP led to attomolar detection levels.^111^ Studies of deep Raman imaging have led to Raman NP detection at depths of 1‐5 cm in ex vivo blocks of tissue, suggesting greatly improved penetration depth *in vivo*.^112^ This information is summarized in Figure 4 and Table 2 **.**

### 5.6 Photoacoustic cell imaging with NP

Photoacoustic (PA) imaging is another optical‐based technique which has recently gained great traction in molecular imaging. PA imaging is considered a mesoscopic imaging approach, because it can be used in microscopic formats to whole‐body formats, and when combined with ultrasound (U/S), enables anatomical, functional, and molecular imaging.^113^ PA imaging can be performed using PA computed photoacoustic tomography (PACT) and 3D scanning tomography (PA microscopy(PAM)). PACT relies on inverse algorithms to reconstruct internal structure. In PA imaging, the target tissue absorbs light and heats up, with accompanying tissue expansion. This expansion results in emission of an U/S signal detected with an U/S transducer. The combination of using near infrared pulses of light, and the lack of background U/S signal, can result in increased depth resolution. Contrast is present due to endogenous proteins (hemoglobin, melanin),^113^ engineered probes (cyanine dyes), and nanomaterials. These nanomaterials include single‐walled carbon nanotubes (SWNTs),^114^ gold nanorods and other gold NP,^115^ and semiconductor particles bearing NIR absorbers.^116^ Studies of cell labeling have demonstrated that silica‐coated gold nanorods (siGNR), exhibit cell toxicity with increasing cell labeling time and concentration. Nonetheless, these studies demonstrated ∼9 × 10^4^ MSC could be detected in the mouse hind limb,^117^ with approximately 100,000 siGNR/cell. In another study, MSC were labeled for 24 hr with 20 nm, citrate stabilized, Au nanotracers, and imaged within the rat calf muscle within a fibrin plug.^118^ This demonstrated that ∼3 × 10^4^ cells could be visualized with photoacoustic imaging. In this latter study, approximately 450,000 NP/cell were present, which may account for the increase in sensitivity. Further studies with cubic shaped, Prussian blue citrate‐polylysine NP demonstrated intracerebral detection levels of 5 × 10^4^ MSC with 40,800 NP per cell. Conversely, PAM approaches demonstrate detection of between 0.5 and 1 × 10^4^ MSC labeled with Au nanocages.^119^ GNR can lose their plasmon resonance effects *in vitro* within the endosome of stem cells, and modification with silica can remove steric hindrance, prevent over‐confinement of particles within endosomes, and improve imaging signal.^120^ This information is summarized in Figure 4 and Table 2.

### 5.7 U/S cell imaging with NP

U/S imaging is a real‐time anatomical imaging tool which is easy to use, safe, and has a high temporal and spatial resolution. B‐mode U/S uses differences in backscattered waves, due to the impedance of tissues, to generate an anatomical image. Here, ultrasonic (mechanical) waves are transduced across the tissue, a backscattered wave is generated and recorded, and an image is generated. Cells cannot be seen using conventional U/S, and contrast is needed. Microbubbles, a gas‐filled bubble with about 5 μm lipid containing shell, are a clinically approved contrast agent, but cannot be used to image cells, as they remain extracellular and in the vasculature.^121^ Scientists first generated U/S contrast by synthesizing silica^122^ and mesoporous NP,^123^ both of which eventually ended up being used for cell labeling and MSC imaging in the context of cell delivery to the heart.^124^, ^125^ These studies report a detection level between ∼7 × 10^4^ and 5 × 10^5^ cells. A recent paper by Chen et al. report using an “exosome‐like” NP for detection limits of 2 × 10^5^ cells experimentally, but report that, in theory, potentially higher levels of sensitivity were possible, with a theoretical limit of ∼5 × 10^2^ cells.^126^ This information is summarized in Figure 4 and Table 2 **.**

### 5.8 CT cell imaging with NP

CT is a technique that helps to visualize differences in tissue attenuation of x‐rays, and it is advantageous because of cost effectiveness, higher spatial resolution, short scan time, and ease of imaging. CT is not considered to have a high sensitivity, and thus has a limited ability to image cells. NP‐based cell imaging with CT is based on the principle that the higher atomic number within its solid‐state structure.^127^ NP composed of gold (Au) demonstrate increased x‐ray attenuation because of their high atomic number (Z = 97). These studies used imaging of prelabeled tumor cells but not stem cells. Surface modification of AuNPs and standard cell uptake assays demonstrate 10's‐100's of picograms (pg)/cell. Schultke et al. showed that using a synchrotron‐radiation approach with focused CT and tomographic imaging and reconstruction techniques, 1 × 10^5^ cells in the brain could be detected, and resolving single cells was possible.^128^ Astolfo et al. also using a synchrotron‐based approach, demonstrated a sensitivity of approximately 1.7 × 10^3^ in a direct injection model AuNP (50 nm)‐labeled cells in the brain.^129^ In both cases described here, synchrotron radiation in a focused beam format is used, which greatly improves spatial resolution. However, it is unclear what the sensitivity is when using small animal imaging instrumentation, such as the microCT, for whole‐body imaging. This topic was recently reviewed in great detail.^130^ This information is summarized in Figure 4 and Table 2.

### 5.9 Single‐photon emission computed tomography cell imaging with NP

SPECT and PET are two important imaging techniques for imaging gamma emitting or positron‐emitting organic or inorganic NP. SPECT imaging is based on radionuclides that emit gamma rays, which can be detected by a gamma camera that has a detector with collimators. The collimators exclude photons that are not directly from the source of the gamma rays. In SPECT, each radionuclide decays which is detected by a gamma camera (single or multihead). Radionuclides either can be directly labeled (^11^C, ^18^F, ^76^Br and ^124^I) or can be chelated (^64^Cu, ^68^Ga, ^89^Zr, ^90^Y, ^99m^Tc, and ^177^Lu) onto a wide variety of NP.^131^ Matching the half‐life of the radionuclide with the pharmacokinetics of clearance of the probe can reduce exposure to the living subject. One of the major advantages of radionuclide labeling is that much lower masses of material are needed to obtain a satisfactory imaging signal compared to MRI or CT. Attachment of a chelator or a prosthetic group to the NP enables binding a radionuclide of interest, as does chelator‐free radiolabeling. This approach has been commonly used for targeting and imaging tumors upon injection, but it is not clear if this approach has been used to label cells and what the cell sensitivity is. SPECT imaging of NP is often in the context of dual labeling, when NP have a primary imaging modality with which they are imaged. This is possible because the surface can be treated for chelation of radionuclides associated with imaging. Thus, dual MRI‐SPECT^132^ and SPECT‐optical imaging^133^ have been actively pursued in the literature. Neural stem cells (1 × 10^6^) labeled with mesoporous NP labeled with DOTA (a chelating agent) bound to 111‐indium were injected into mouse brains with glioblastoma and imaged with SPECT.^134^ This information is summarized in Figure 4 and Table 2 **.**

### 5.10 Positron emission tomography cell imaging with NP

In PET, the positron emitting radionuclide annihilates a nearby electron (100 μm) and emits two, anti‐parallel, high energy, 511‐keV gamma photons, which are detected in a coincident fashion. The drawbacks of PET are that the spatial resolution is low (mm), it is highly specialized and can be costly. A wide range of NP have been labeled with positron emitting radionuclides such as 18‐F and 64‐Cu, and these have recently been reviewed,^135^ but it is unclear if these PET‐labeled NP have been used for stem cell imaging. This information is summarized in Figure 4 and Table 2.

### 5.11 Summary

A summary of all the imaging modalities and NP used is in Figure 4. In this section of the review, we have summarized a wide range of NP imaging techniques, noting their strengths and weaknesses. Furthermore, we have shed light upon the numerous types of NP used and some structural features of these NP of interest to the chemical and bioengineer. Finally, we have reviewed when possible, any data focused on cell imaging and cell sensitivity, in which stem cells (or cancer cells) have been prelabeled with the corresponding NP, injected in a small animal and imaged. These studies indicate that prelabeling can be used to image stem cells at reasonable cell numbers. However, we did not see limit of detection studies in most cases, which suggests there is opportunity to do that. Many variables affect prelabeling, including concentration of NP per cell, duration of labeling, transfection reagent used, and cell type. Imaging parameters and host parameters may also affect imaging signal. Although these approaches have not necessarily contributed to our knowledge of stem cell biology, one can argue that cell prelabeling for imaging a population of cells after initial injection can be used to optimize and localize the initial aspects of cell therapy. Using a highly sensitive modality for NP labeling combined with an imaging modality with a high spatial resolution may provide further insight into the exact location of the injection, which can be used to further optimize cell delivery strategies.

## 6 CELL IMAGING WITH REPORTER GENES

### 6.1 Overview on RG

Reporter genes (RG) are a more widely applicable tool for cell imaging compared to NP, as not only can cells be imaged *in vivo*, but other aspects of cell biology can be studied noninvasively *in vivo*.

RG encode for genetically encoded proteins, often enzymes, which are selectively driven by a promoter of choice. RG are expressed within a cell of interest as part of a construct that is either transiently transfected using viral (adenovirus) or nonviral techniques, or is inserted as a transgene using viral (lentiviral, retroviral) or nonviral techniques, or as a knock‐in at a particular gene locus. The promoter, which drives RG, determines the specific biological event which can be observed, whether the desired application is cell tracking, cell growth, or cell differentiation. RG can emit an imaging signal which can be detected noninvasively *in vivo*. RG are genetically encoded, and therefore they are not diluted with cell division, as would occur in NP labeled cells. Very often, the RG interacts with a probe of interest, which enables generation of the imaging signal. In general, the RG may be toxic or affect biology adversely, or may be affected by the cell themselves. The half‐life of the RG is critical, and the shorter the half‐life, the more that the signal is representative of the actual biological event. Below we will review key RG and how they have significantly contributed to molecular imaging approaches toward cell imaging.

### 6.2 Clonality in RG‐containing cells

As mentioned earlier, exogenous RG can be delivered, as single or multiple copies, to stem cells via viral (lentivirus, retrovirus) or nonviral (plasmid)‐based techniques, which are well established. Single clones can be expanded, such that the cell population itself is clonal, meaning that each cell has the identical numbers of transgenes per cell at the same loci. Alternatively, polyclonal cell population can be utilized, in which each cell has “approximately” the same number of RG copies per cell, but each cell may have RG copies at different locations from each other. Depending on the application for imaging, each avenue is possible, and has advantages and disadvantages.

### 6.3 Fluorescent proteins

The discovery,^136^ cloning,^137^ and development of green fluorescent protein (GFP)^138^ opened up a new field by enabling a wide range of biological processes that could be monitored noninvasively, for applications ranging from cell labeling, studying promoter activity, creating transgenic organisms, intracellular sensing, protein‐protein interactions, nucleic acid labeling, and so forth.^139^ This ancient metazoan gene, when expressed, folds into a ß‐Barrel that contains a chromophore that is generated by cyclization and oxidation of Serine‐Tyrosine‐Glycine (Ser‐Tyr‐Gly) amino acids.^138^ Mutants of GFP have been engineered to generate a family of fluorescent proteins, and these can be compared by brightness, which is a product of the extinction coefficient and the quantum yield. Despite many positive aspects, a drawback of visible light‐emitting fluorescent proteins is that their wide emission spectrum is not in the NIR range. Thus, they can be widely applied to *in vitro* assays, but *in vivo* imaging is limited to specific applications. Intravital microscopy, which has an extremely high lateral spatial resolution and high sensitivity, matches nicely with the use of visible light‐emitting fluorescent reporter genes for asking biological questions about stem cells. Further development of red‐shifted fluorescent proteins^140^ will enable NIR emission and improved *in vivo* whole body and intravital imaging of stem cells. Along these lines, mCardinal (emission peak 659 nm) expressed within muscle stem cells has been utilized to demonstrate *in vivo* differentiation of muscle.^141^

### 6.4 Fluorescent proteins and IVM and MPM‐based cell imaging

IVM, two photon and MPM, together with RG technology, have been used to learn a great deal of information about stem cells in recent years. These systems often require tissue preparation with a window, often analyzing an ectopic or orthotopic site within hard tissue (i.e., bone) or soft tissue, or on orthotopic tissue flaps of interest. The window enables serial imaging at various temporal resolutions (seconds to days). MPM enables improved depth penetration and spatial resolution compared to IVM and relies on the co‐excitation of a fluorescent protein/fluorophore with multiple lower energy (and higher wavelength) photons that simultaneously stimulate the fluorophore. Because co‐excitation is a rare event, a photon flux illuminated within the field of view is necessary. The field of view of these approaches is a function of the microscopic objective used and can vary between centimeters to micrometers of tissue. Combining IVM/MPM for studying stem cells has commenced the last 10‐20 years, and a wide variety of tissue systems have already been investigated, including HSC,^142^, ^143^, ^144^, ^145^ other blood forming progenitors,^146^ intestinal stem cells,^147^ skin/hair stem cells,^148^, ^149^ mammary stem cells,^38^, ^150^ germ stem cells,^151^, ^152^ MSC,^153^, ^154^, ^155^ muscle stem cells^141^ dendritic cells,^156^ cardiac stem cells,^157^ and neural stem cells.^158^

The advantage here is that ASC, which are difficult and at times impossible to culture, can be studied in their intact environment. Furthermore, the response during regeneration, after injury, to disease, and so forth can be studied noninvasively using these approaches. It is important to note that, in some studies using IVM/MPM, the RG was expressed in the stem cell itself, while in other cases, a supporting cell or tissue structure expressed the RG. A dizzying array of biological fates or mechanisms have been explored using these techniques, including homing, trafficking, interstitial transport, differentiation, migration, stem cell‐niche interactions, asymmetric versus symmetric cell divisions, stem cell heterogeneity, tissue homeostasis, spatial organization of the niche, differential growth, collective cell movements, and so forth. Importantly, not only can IVM/MPM enable single cell level imaging but also enable whole tissue/organ imaging. This versatile imaging tool thus facilitates concepts of tissue mapping, imaging differential tissue growth, imaging tissue regeneration, and imaging organ/tissue development.^38^, ^159^ Not only have ASC and the ASC niche been imaged using these approaches, but also CSC‐mediated processes, like tissue remodeling and migration have been studied using IVM and MPM.^38^, ^150^, ^160^ This has proved valuable, as the CSC and non‐CSC are believed to have different functions, and these functions have been elicited using the capabilities of IVM/MPM.

**Figure 5:**
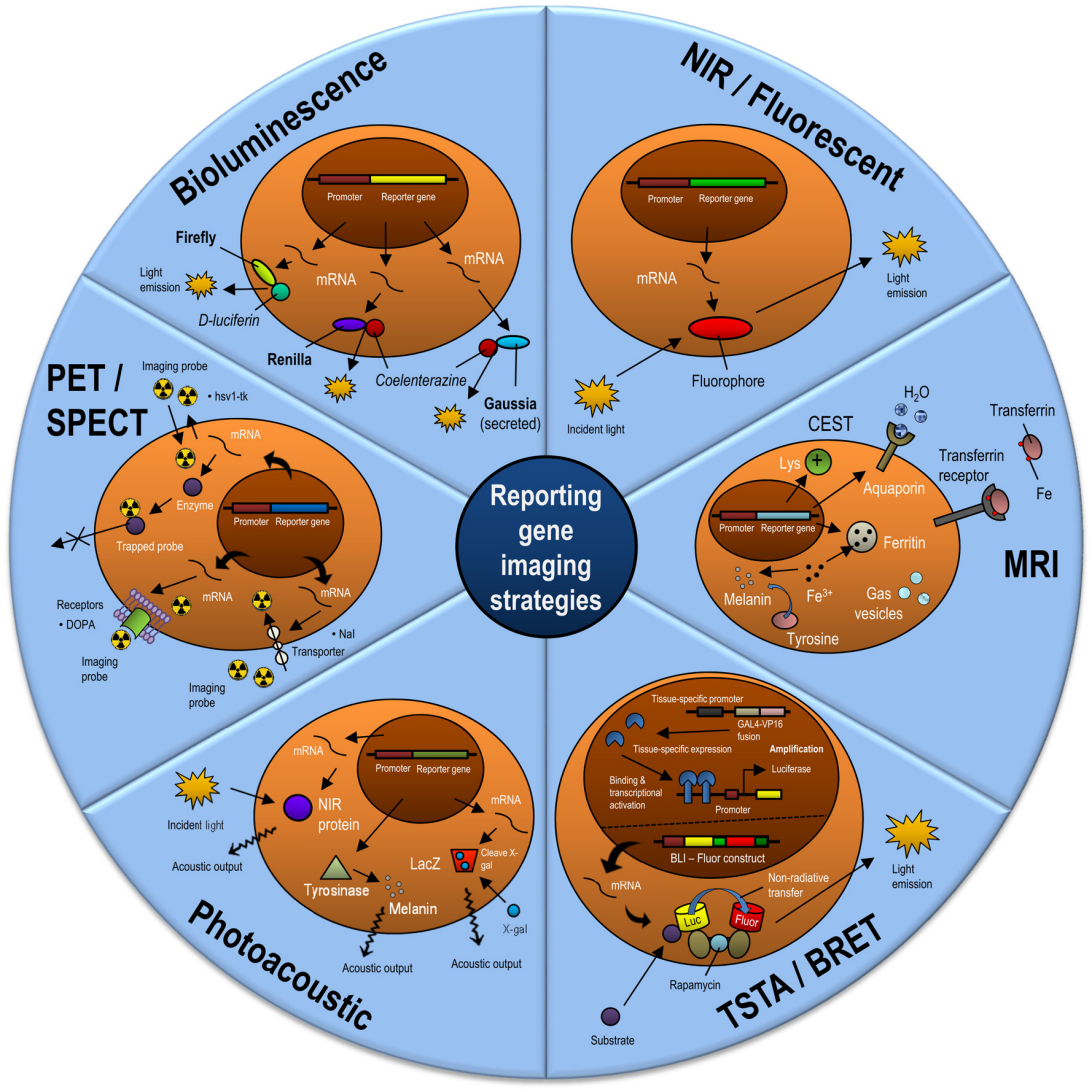
Reporter gene‐based imaging strategies. While NP‐based strategies are primarily focused on cell labeling, many reporter gene strategies have been used to understand underlying biology in addition to imaging cells in vivo. RG strategies for in vivo imaging include near infrared (NIR) and fluorescent RG, MRI RG (ferritin, transferrin, CEST, Tyrosinase, gas vesicles, aquaporin), photoacoustic (Lac Z, Tyrosinase, NIR proteins), PET/SPECT (hsv1tk, dopamine receptor, and sodium iodide transporter), and bioluminescence (Firefly luciferase (Fluc), Renilla luciferase (Rluc), and Gaussia luciferase (Gluc). In each modality, we demonstrate how the RG is expressed and how an imaging signal is generated

While the results have continued to shed new light on the biology of stem cells, there are challenges with these approaches. A simple point is, how much can we generalize about all the niches within a tissue of interest, by examining one niche in several mice or even a few per mouse? A second point is, what effects does a window preparation have? While control experiments can prove that the window preparation has effects, it is highly likely that window might alter the transport, microcirculation, and biomechanical environment, and the host (mouse). Limitations to the technique include imaging only 150‐450 μm (superficial) locations, or prespecified locations (in the vicinity of bone to drill in the optical window), while deep tissue and soft tissue imaging remain a challenge. Stem cell niches have several targets, including the stem cells, niche supporting cells, vasculature/perivasculature, and extracellular matrix. To address this, a seminal study employed long term dye to label HSC, an osteoblast‐restricted 1a collagen promoter to image osteoblasts, second harmonic generation to image collagen, and nontargeted NIR dots to image vasculature.^142^ The advantage of this approach was that the scientists were able to observe and quantitate HSC homing and single cell divisions to the periosteal and perivascular niche within the mouse calvarium.^142^ Pushing the technology forward, there remain many questions about the stem cell niche, particularly from a chemical engineering point of view. What are the states of the niche? How can the boundaries of the niche be determined exactly, throughout the tissue? What are the concentration of key soluble factors within the niche, in terms of local concentrations near the vicinity of the stem cell? MPM/IVM may continue to provide some clues. This information is summarized in Figure 5 and Table 2.

### 6.5 Bioluminescent RG‐based cell imaging

Bioluminescence imaging (BLI) with RG has been a tremendous development, which has greatly impacted molecular imaging and *in vivo* imaging of stem cells. In contrast to fluorescent reporter genes, BLI imaging has no background signal. Furthermore, excitation is not needed, as light is emitted from a chemical reaction with a substrate. Light levels are lower than fluorescent proteins, and the technological development of highly‐sensitive, cooled charged couple device (CCD) cameras have enabled imaging despite these low light levels. BLI has high sensitivity, although this decreases proportionally to depth, and low spatial resolution, even though many tissue and organ locations can be distinguished. BLI is often combined with X‐Ray or CT to superimpose the molecular image on top an anatomical image. For BLI imaging to be implemented, Firefly luciferase (Fluc), cloned from fireflies and expressed in mammalian cells, together with technological developments for imaging, needed to be established.^161^ Fluc oxidizes D‐luciferin to peroxy‐luciferin, in the presence of O_2_ and cellular ATP. While reaction intermediates are generated, they produce light in the range of 550‐700 nm, and light at 650 nm and above can penetrate the NIR window for *in vivo* imaging. Renilla luciferase (Rluc), isolated from the sea pansy, was also developed for *in vivo* imaging.^162^ The enzyme Rluc catalyzes coelenterazine oxidation leading to bioluminescence. Coelenterazine consists of an imidazolopyrazine structure {2‐(p‐hydroxybenzyl)‐ 6‐(p‐hydroxyphenyl)‐8‐benzylimidazo [1,2‐ a]pyrazin‐3‐(7H)‐one} that emits light in range of 400‐650 nm, with a maximum at 480 nm. Rluc is a smaller monomeric protein compared to Fluc (36 vs. 61 kDa), has less cofactor dependence (oxygen only), and has more rapid signal kinetics. Rluc has been engineered extensively, including for enhanced stability in serum,^163^ and red shifting^164^ for improved *in vivo* imaging. This enables increased light emission within the NIR window. For *in vivo* imaging with BLI, a substrate is required that has to be stable, bioavailable, have favorable pharmacokinetics. Fluc has been used for a wide range of cell imaging applications, including neural stem cell tracking in the brain,^158^ cardiac cell transplantation,^165^, ^166^ assessment of gene delivery,^167^ epigenetic modulation of reporter expression,^168^ immunosuppression efficacy,^169^ graft versus host disease,^170^ evaluation of tissue scaffolds,^171^ whole body HSC reconstitution^172^ liver cell therapy,^173^ encapsulated *in vivo* cell viability,^174^ decellularized liver matrix,^175^ oxidative stress within transplanted cells,^176^ differentiation,^177^, ^178^, ^179^ and multimodality imaging.^180^ This information is summarized in Figure 5 and Table 2 **.**

### 6.6 Transgenic mice and promoter engineering for cell imaging

For RG used *in vivo*, the promoter plays a significant role, in that it dictates the constraints under which RG are expressed. For cell tracking applications, donor cells from a transgenic mouse, most commonly the L2G85 mouse, can be used. The L2G85 mouse bears a transgene (homozygous) that is composed of human cytomegalovirus (CMV) immediate early promoter enhancer with chicken beta‐actin/rabbit beta globin hybrid promoter, or the (CAG) promoter, which drives a fluc‐egfp fusion protein.^172^ Because all the cells in the mouse express RG strongly, these transgenic mice have been actively used as donor mice in cell transplantation experiments.^172^ For engineering stem cells of interest with RG prior to transplantation, ubiquitous human promoters in human cells are valuable, such as ubiquitin C.^181^ Although CMV is a widely used ubiquitous promoter, and it likely has the strongest activity and would lead to the lowest sensitivity for *in vivo* imaging, studies have demonstrated that CMV can be methylated *in vivo*.^182^ Many questions arise when working with either ASC or hPSC‐derived cells, including viability, homing, engraftment, and proliferation, all of which can be answered using constitutive promoters. Differentiation, on the other hand, requires strategies to engineer promoters and/or RG. Most differentiation promoters are too weak to see a change in signal over time.^183^ We recently engineered a two reporter approach in which constitutive activity was measured with ubiquitin C and Fluc, while differentiation was measured with a cloned promoter and Rluc.^178^ In this case, the differentiation promoter was Oct4 and was strong initially, but then was shut down during differentiation, although we observed complex kinetics that had previously not been appreciated.

### 6.7 Improving deep tissue cell imaging

New strategies are continually needed for deep tissue imaging of cell differentiation (Figure 5). The weakness of the promoter itself, combined with deep tissue imaging, which imposes more scattering and absorption of light, makes bioluminescence and fluorescence particularly challenging. Fortunately, strategies have been developed to address the weakness of differentiation promoters. One strategy is termed the “TSTA” or two step transcriptional activation, which essentially involves synthetic biology techniques to build a new gene circuit, an “amplifier” within the cell of interest.^184^ In this system, the differentiation promoter, instead of driving the differentiation gene, drives the expression of Gal4‐VP16 fusion protein. Next, the reporter is driven by 4‐5 Gal4 binding sites. As a result, Gal4‐VP16, an unusually potent transcriptional activator,^185^ activates transcription of the reporter, rather than the original promoter. These initial studies demonstrated a 50‐fold increase in Fluc *in vitro* expression, and a 5‐fold increase in *in vivo* BLI signal. This TSTA system has been used to enable visualization and enhanced imaging signal in the setting of T cell differentiation,^183^ imaging therapeutic gene expression,^186^ and stem cell differentiation.^187^ Considering that stem cell differentiation is a complex process that may involve transitions between an initial state and several transitional states, new strategies will be needed to image multiple differentiation states.

A second approach for deep tissue imaging is bioluminescence resonance energy transfer (BRET) strategies. BRET strategies were initially developed in the context of analyzing protein‐protein interactions *in vivo*. Fluorescence resonance energy transfer (FRET) and BRET involves the nonradiative transfer of energy between the donor and acceptor molecules by the FÖRSTER mechanism, in which energy from a donor chromophore is transferred to an acceptor chromophore through nonradiative dipole‐dipole coupling and has a radius‐dependence of 1/r^6^. BRET technology uses a fluorescence and bioluminescence protein pair. Here, a bioluminescence substrate is added to the living subject, exciting the bioluminescent protein to luminesce. This transfers the energy, nonradiatively, to the fluorescence protein. Next, the fluorescent protein emits energy that reflects an interaction between the pair of proteins, often due to protein‐protein interaction. Manipulation of the donor protein, together with red‐shifted acceptor fluorescent protein, and a new substrate that produced red shifting^188^ have led to improvements in the ability to image 3 × 10^6^ tumor cells entrapped and spread throughout the lungs (deep tissue) after tail‐vein injection of cells. However, thus far, these BRET systems have not been used to image stem cells in deep tissues, but this would be valuable for imaging stem cells within internal organs like the intestines, liver, pancreas, and lungs. This information is summarized in Figure 5 and Table 2 **.**

### 6.8 Radionuclide‐based (PET and SPECT) reporter genes for cell imaging

PET RG are another class of RG and are the only RG system that has been used to image therapeutic genes and therapeutic cells not only in small animals and large animals but also in patients. The value of PET is its high sensitivity, and importantly, equal sensitivity at all depths (tomographic). Furthermore, PET enables data from a small animal, preclinical model to be translated to clinical PET studies in patients. The development and use of PET RG intersects across several disciplines, including the biology of the RG, radiochemistry/radiolabeling/ pharmacokinetics of the reporter probe, and the generation of the imaging signal.

The first PET RG, involving the herpes simplex virus type I thymidine kinase (hsv1‐tk) RG and radiolabeled PET reporter probes, became an ideal model for considering the requirements for a PET RG.^189^ The list of requirements include: (a) that the RG should be a mammalian protein (to evade the immune response), (b) the RG should be specific in in its interaction with reporter probes, (c) there should not be significant accumulation in cells without RG, (d) The reporter probe should be stable *in vivo* and ideally not converted to complex metabolites, (e) reporter probe should be rapidly cleared from blood and nonspecific tissues and have an elimination route that does not interfere with signal detection, (f) the reporter probe should be able to be easily radiolabeled without changing its properties, (g) the reporter probe and its metabolites should not be cytotoxic in vivo*,* (h) The size of both the promoter and the RG should be small enough to be cloned into a delivery vehicle (i.e., lentivirus) (This requirement is not important in regards to generating a transgenic organism), (i) the reporter probe must be delivered to the target location without the cell membrane acting as a significant barrier, (j) the reporter probe should correlate with levels of the RG, including mRNA levels and protein levels, over a range of relevant concentrations, and (k) if the RG is reporting for an endogenous gene, the RG and the reporter probe should correlate well with the levels of the endogenous gene, including mRNA and protein levels.

One of the first PET RG developed was the result of studies of a drug (small molecule) receptor combination which was already in place in the clinic. Hsv1‐infected cells express hsv1‐tk, and the corresponding protein phosphorylates the drug acyclovir. Subsequent phosphorylation of acyclovir monophosphate by guanylate cyclase forms acyclovir diphosphate, which is subject to various cellular kinases and leads to the formation of acyclovir triphosphate. Acyclovir triphosphate then leads to chain termination through incorporation into DNA and inhibits viral DNA polymerase, both of which stop the infection. Tjuvajev et al.^190^ realized that this system could be used to track and kill brain cancer (glioma) cells. Tjuvajev et al. showed that if the derivative of acyclovir was radiolabeled with fluorine (FIAU), and the tumor cells expressed hsv1‐tk, then the radiolabeled drug, when given at low (trace) levels, would accumulate in tumor cells. This was used to image the cells via autoradiography. Similarly, Gambhir et al. radiolabeled the drug ganciclovir, used it to image adenoviral‐mediated hsv1‐tk gene expression in the liver, and the first PET images of this system were generated. The correlation of gene expression, protein expression, and PET imaging signal (% injected dose per gram) demonstrated that this PET RG system was quantitative. A second PET RG system involves radiolabeled PET dopamine‐based ligands that were developed for the dopamine receptor (D2R), which normally is on brain striatum and pituitary glands.^191^, ^192^ Regarding the hsv1‐tk system, scientists improved expression levels by using mutant enzymes with improved reporter probe uptake and imaging signal,^193^ identifying an improved reporter probe 9‐(4‐18F‐fluoro‐3‐[hydroxymethyl] butyl)guanine (18F‐FHBG), and testing 18F‐FHBG pharmacokinetics and safety profile in patients.^194^ This system was shown valuable for cell imaging in the setting of cardiac cell transplantation.^165^, ^195^ Importantly, this sr39tk‐FHBG combination was tested in patients in a trial of gene therapy directed toward liver cancer,^196^ and in the setting of T cell therapies expressing HSV1‐tk in patients.^70^, ^197^ To determine the sensitivity of cell imaging in patients, we recently performed a study of MSC injection in swine hearts, followed by MRI and PET.^82^ We found the detection levels in the heart, with the HSV1‐tk sr39tk mutant, and 18F‐FHBG, to be ∼2.5 × 10^8^ cells. Although other studies did not perform a full, quantitative limit of detection study, reports have ranged between 50‐fold and 2‐fold less than what we reported.^198^

The sodium iodide symporter RG is a third reporter system, which uses a cell membrane transporter as the RG and radiolabeled iodide as the probe of interest.^199^ In this RG system, radioactive iodine is injected and selectively accumulates in cells that express the iodine symporter. The iodine symporter is normally present in thyrocytes in the thyroid gland for selective iodine transport in the process of thyroid hormone synthesis within the thyroid follicle. About 7.5 × 10^6^ cells have been grown for 4 weeks and imaged using planar scintigraphy for ^123^‐I.^199^ The advantage of this system is the complex aspects of PET radionuclide synthesis are not required, and a second advantage is that radiotherapy can be used to destroy labeled cells in the setting of cancer therapy or suicide therapy. Although studies demonstrate comparable uptake to the HSV1‐tk system, the limit of cell detection is unclear. This information is summarized in Figure 5 and Table 2.

### 6.9 MRI reporter genes for cell imaging

As MRI is a potent and widely used noninvasive imaging technique, MRI reporter genes, provided they are able to increase sensitivity, can be a valuable tool for the *in vivo* imaging of stem cells in patients. The first MR reporter gene involved the enzymatic cleavage of a probe termed EgadMe, which chelates gadolinium, with protection by a galactopyranose. When galactopyranose is cleaved enzymatically by beta‐galactosidase, then the gadolinium is released and results in enhanced MRI contrast.^200^ Engineered transferrin receptor is an approach in which iron and iron particles can be shuttled into cells for enhanced uptake, change in relaxivity, and enhanced imaging on T2* weighted images.^201^ Transplantation of tumors with the engineered receptor, followed by injection of 3 mg of 3 nm SPIO NP, demonstrated a ∼25%‐fold change in signal to noise ratio. Increased transferrin receptor was used in an MSC study, but no change in endogenous signal was observed.^202^ Ferritin protein has been used as MRI reporter gene as a way of manipulating iron homeostasis within the cells.^203^ These studies demonstrated an ∼10% increased relaxation time in transplanted tumor cells that conditionally expressed ferritin. In the setting of cardiac cell transplantation into the heart, MRI signal loss correlated with histology, demonstrating the potential feasibility of using this approach for stem cell tracking.^204^ A third type of reporter is a chemical exchange saturation transfer (CEST). Here, lysine‐rich proteins bear amide protons which exchange with water protons, generating a MRI signal change.^205^ CEST reporters in tumors placed within the brain showed a 4‐fold increase in signal intensity. Importantly, CEST reporters may have multiplex capability, because the radiofrequency signal needs to be tuned to the particular protein used. A fourth type of MRI RG involves gas‐filled vesicles. When these gases filled vesicles, expressed within bacteria, are filled with hyperpolarized Xenon (^129^ Xe), they have been shown to generate 100‐fold to 10,000‐fold improved signal and represent fundamentally new MRI RG.^206^ A fifth type of MRI reporter is one in which the diffusion of water has been altered, by the expression of aquaporin, which results in enhanced contrast on a diffusion weighted MRI due to altered diffusion of water.^207^ This information is summarized in Figure 5 and Table 2 **.**

### 6.10 Photoacoustic reporter genes for cell imaging

Photoacoustic (PA) RG are an active field of research leading to an explosion of research in the area of photoacoustic imaging. The first report of cell imaging of RG using PA was when 5 × 10^6^ lac Z‐expressing tumors were imaged beneath the scalp of rats after injection of X‐gal, a substrate for Beta galactosidase which is expressed by the lac Z gene. The imaging time, however, was 25 min. Here, X‐gal is cleaved to galactose and 5‐bromo‐4‐chloro‐3‐hydroxyindole, which then dimerizes and is oxidized to 5,5′‐dibromo‐4,4′‐dichloro‐indigo.^208^ Tyrosinase RG were evaluated and demonstrated a PA signal when expressed in nonmelanin containing cells. Tyrosinase (Tyr) is a rate limiting step in melanin production, and melanin is a pigment in the skin, hair, and eye. Tyr‐expressing cells were imaged by PA with an estimated 45 cells at 3 mm in depth,^209^ while another study^210^ reported an *in vitro* imaging sensitivity of 2.5 × 10^3^ cells. In this latter study, at least 1 × 10^7^ Tyr‐expressing tumor cells were imaged using PA, but an *in vivo* limit of detection study was not performed. Other studies also have produced cell imaging of 5 × 10^6^ tyrosinase expressing cells *in vivo*, and 1 × 10^6^ cells implanted at 6‐8 mm were clearly visualized.^211^ In contrast, fluorescent proteins are not ideally suited as PA genetic reporters because of their low extinction coefficients, poor PA generation efficiency, and a lack of variants with a 650 nm emission. NIR fluorescent protein‐derived from bacteria phytochrome photoreceptors (BphPs), called BphP iRFP713 protein, or iRFP, has been shown be detectable by PA *in vivo* due to its high extinction coefficient and low quantum yield, and the fact that it's absorption and emission spectrum lie in the NIR range.^212^ Filonov et al. showed that 1 × 10^6^ iRFP‐expressing breast cancer cells grown for 2‐3 weeks may be imaged at 280 μm lateral and 75 μm axial resolution, and a depth of 4 mm. However, iRFP proteins require endogenous biliverdin to become fluorescent and may suffer from bleaching and transient absorption intermediates.^213^ Overall, PA RG are an extremely active area of research and have recently been reviewed in detail.^214^ This information is summarized in Figure 5 and Table 2 **.**

### 6.11 Secreted reporters

In many instances, one would want to detect the presence of the RG, but knowing the exact location may not be as critical. In this case, constitutive promoters drive secreted reporter RG. The advantages of using a secreted reporter are that repeat, noninvasive measurements can be made from the body fluids, at high temporal resolution, and the animal does not need to be sacrificed or repeatedly anesthetized for extensive periods of time as they would need to be with serial imaging. Thus, secreted proteins also qualify as noninvasive monitoring. If the promoter is exchanged for another promoter (differentiation) then many other aspects of cell fate can be monitored. The secreted protein also should not mount an immune response when entering body tissue and fluids. Also, it should not irreversibly or adversely interact with proteins in the blood. Secreted reporters are essentially reporter proteins that have the appropriate molecular signals (signal sequence) to be secreted. A secreted reporter must be stable in the blood and have a relatively long half‐life. Furthermore, there should be an assay that enables the probe to be easily detectable, and the signals must be proportion to the number of cells that are present over several orders of magnitude. Two secreted proteins that have been used are human placental secreted alkaline phosphatase (SEAP) and gaussia luciferase (Gluc). SEAP is a heat‐stable, modified human 64 kDa protein, which is normally a placental cell surface membrane protein, but has been modified to be secreted. Importantly, SEAP levels *in vitro* have been shown to be correlated *in vivo* to the amount of SEAP delivered nonvirally^215^ and sensitivity of the assay is 50 pg/mL.^216^ Mouse SEAP (mSEAP) has been engineered to avoid immune responses in murine models and has been shown to correlate with cell number and tissue growth of transplanted cells.^217^ The SEAP assay however takes time and can be limiting in high throughput assays. Gaussia luciferase (Gluc) is a 480 nm emitting luciferase which oxidizes the substrate coelenterazine. Because the protein is small (19.9 kDa) and luminometry is highly sensitive and quantitative, the assay for Gluc is easy, fast, and highly sensitive (1000‐fold more than SEAP). It has been widely used as a secreted protein for many applications in therapeutic monitoring. In one study, tumor cells constitutively expressing Gluc were implanted at various cell numbers and were imaged with bioluminescence imaging. Furthermore, samples of blood and urine were collected and an ex vivo assay for Gluc was performed. This study showed that Gluc in blood and urine was linear with cell number and correlated with *in vivo* imaging of cell number.^218^ Despite its wide usage, Gluc has not yet routinely used for *in vivo* tracking of stem cell fates. This information is summarized in Figure 5 and Table 2 **.**

### 6.12 Summary

RG offer the ability to genetically encode protein that ultimately generate an imaging signal and are summarized in Figure 5. For stem cell‐ and tissue engineering‐based therapies that are focused on large amounts of cell and tissue replacement, there is a need to monitor these cells in patients. Furthermore, in small and large animal preclinical models, there may be many opportunities to develop reporter‐based assays that are relevant for regenerative medicine. These can involve further understanding of specific cell‐cell, cell‐matrix, or cell soluble interactions of stem cells within the niche, or understanding of cell‐host tissue interactions. RG have been successful for tracking cells in small animals, large animals, and patients, but next generation RG can potentially focus on emerging imaging techniques or on assay development for scientists within the field of regenerative medicine. Furthermore, several orders of magnitude of improvement sensitivity are necessary for MRI and PET RG will improve the clinical utility of these approaches, such that stem cell therapies are further advanced. In our final section of this review, we will consider what next generation regenerative medicine can be.

## 7 “NEXT GENERATION” REGENERATIVE MEDICINE

Currently, the goal of cell and tissue‐based therapies is to replace damaged or dysfunctional tissues. As many of these approaches have not yet been translated to patients, it would be useful to highlight how advanced molecular imaging strategies and tools may be used to improve regenerative medicine‐based approaches or improve the translation of these approaches into patients.

The standard in the field is to use tissue sections to assess regenerative status. However, these are endpoint assays, which require animal sacrifice in preclinical models or biopsy in preclinical models or patients. These biopsies and their analysis are often not quantitative. Furthermore, as the tissue is being processed there can be a loss of information, and differences between individual subjects can be lost. Based on these concepts, below we discuss various ways, other than cell imaging, in which imaging can improve cell and tissue based studies. Figure 2 summarizes the variables when performing cell or tissue therapy which may influence outcome, and these areas represent opportunities for new imaging approaches.

### 7.1 Integrating imaging with *in vivo* regenerative medicine assays

A simple area where molecular imaging can improve regenerative medicine potential is an improvement of *in vivo* ASC assays, reviewed in section 2.2. Stem cell assays involve transplantation of ASC into cleared tissues and are sometimes called a regeneration assay. Assay analysis is based on tissue sections is qualitative, although flow sorting of the tissues is quantitative, and both assays are endpoint assays. *In vivo*, noninvasive molecular imaging could investigate growth/regeneration at earlier time points in the assay, at lower cell numbers, and more quantitatively. In our studies of IVM, applied to mammary development, mammary stem cell regeneration, and cancer stem cell growth, we discovered several new findings, simply by incorporating *in vivo* imaging^38^ into standard assays. While current assays can analyze bulk populations and standard endpoints, *in vivo* imaging can help obtain information based on serial imaging, and provide real time (temporal and spatial) information regarding a stem cell assay. The use of imaging also enables each mouse to serve as its own control, and could improve data and statistics. Small sources of anatomical and physiological variation can lead to variability in a cell therapeutic response. Using imaging to quantify this with improved controls may lead to more accurate observations and conclusions, determining which research directions are more promising. *In vivo* imaging may also help identify rare events which cannot be obtained by traditional assays, and as stem cell self‐renewal and asymmetric divisions are rare events, developing ways to observe this *in vivo* can improve our knowledge of these processes. This imaging data can also be used to build quantitative silico models of tissue growth and predict growth changes due to intrinsic and extrinsic perturbations.

### 7.2 The interactions between cells and tissue microenvironment

Whether ASC, MSC, or hPSC‐derived cells are being used therapeutically, there is still a central question regarding how stem cells affect the tissue microenvironment *in vivo*. If the addition of cells to a tissue is viewed more as a perturbation, further questions arise: (a) How does the stem cell or tissue transplantation effect the endogenous tissue hierarchy, tissue homeostasis, and the various aspects of the tissue microenvironment into which it was transplanted? (b) in terms of tissue hierarchy, how does an ASC find its niche? (c) does an ASC compete with endogenous stem cells, or does it create its own niche? (d) If it is a progenitor cell, or a mature cell, does it find its place in the hierarchy, and if so, how? (e) are the transplanted stem cells fully functional? (f) how is extracellular matrix organization affected? (g) how are the neighboring cells affected by therapeutic cell transplantation? (h) If still present, does the diseased tissue reverse its state, or does it die in a competition with healthy cells? We often assume that stem cell transplantation or tissue engineered constructs may remove a disease state, but we can use imaging to understand this question at a deeper level. The simplest approach to image the interactions between cells and tissue environment is to employ IVM at small scales, or to add an anatomical imaging technique, like MRI or CT, to a highly sensitive molecular imaging technique like BLI or PET. Using these approaches, one can track the cells, image the microenvironment, and begin to perhaps determine the relationship between cell and the microenvironment.

To image tissue hierarchy, methods to employ multiple RG, or even methods that use multiple probes that interrogate the tissue hierarchy, are needed. The approaches of solid or hollow epithelial organs might be different (liver, breast, intestine) compared to systemic organs like the bone marrow. Finding methods to interrogate the contents, state, and location of stem cell niches noninvasively in these tissues would be valuable. For example, information about the niche or tissue environment could be used to guide delivery and type of cell therapy. Perhaps knowledge about tissue hierarchy or niches could be used to understand regenerative states in patients that predict diseases states prior to cell or tissue therapy. If these aspects cannot be measured using new molecular imaging approaches, then perhaps what is needed is again, an in silico modeling approach to help predict, visualize, and model how tissues behave under particular conditions.

### 7.3 Interrogating tissues prior to cell transplantation

With the continued development of regenerative medicine, for a particular regenerative injury, there will be several options (i.e., ischemic heart disease, Figure 2). An important question will be, how does one choose the correct regenerative medicine treatment option? Currently, this is determined by trial and error in clinical trials, averaging across large groups of patients. For example, in cardiac therapy, skeletal myoblasts, C kit^+^ cardiac stem cells, Sca1^+^ progenitor cells, MSCs, and hPSC‐derived progenitor cells are all used, including imaging (cardiac echo), function (cardiac catherization), and blood tests (coagulation profile). As cells are being placed within the heart, it would seem that molecular factors of the local heart tissue could dictate the success or failure of the therapy. Thus, one can conceive that molecular imaging tests or molecular diagnostics could be developed to reveal molecular information. Recently, Jokerst et al.^219^ identified molecular biomarkers that are associated with patients that respond to cardiac therapy. Perhaps molecular imaging tests of not only the blood, but also the tissue, can also be used to predict cell therapy. These can potentially include molecular probes which identify angiogenesis, metabolism, inflammation, or other molecular aspects of the milieu that might predict successful therapy. Other host factors, including aspects of the local transcriptome and proteome, may heavily influence the outcome of cell therapy. Thus, molecular imaging can potentially be used as a diagnostic prior to cell therapy.

### 7.4 Translating cell therapy to patients and multimodality imaging

A major problem is translating results from small animals to patients. For example, there have been many successful cell therapies^220^ in small animals, but how can this be translated to patients? Dosing with pharmaceutical can be done based on mass of the patient (or the amount of receptor available), but because molecular targets of cell therapies are complex, it is unclear how to perform exact dosing. In a recent set of publications, we demonstrated experimentally an idea of how molecular imaging in small animals and large animals could be connected, to improve the clinical translation of cell therapies.^82^, ^180^ In this case, multimodality RG, bearing eGFP (enhanced GFP), Fluc2, and hsv1 mutant (sr39tk), for fluorescence, bioluminescence, and PET RG imaging, were transduced into MSC. These MSCs were tested in small animal disease models and Fluc signal was associated with cell survival.

An endpoint of 14 days postmyocardial infarction was used, and MSC imaging demonstrated this endpoint was achieved. Because these MSC had reached the appropriate criteria and they also expressed the PET RG (fusion protein) under the same promoter, the same exact MSC cell line was tested with PET imaging in a large animal model. In this manner, one could imagine that a series of therapeutic cell candidates may be tested in a small animal model, and only the therapies that reach the specific molecular imaging endpoints could be tested in the large animal model. Furthermore, signals due to dosing in small animals (BLI) could be compared to signals in large animals (PET RG) to understand dosing‐related issues.

### 7.5 Summary

In this final section, we have summarized potential ways molecular imaging can be used to further inform regenerative medicine, in what we call next generation regenerative medicine. We predict that a wide range of imaging modalities and tools will continue to increase within molecular imaging for applications like cancer. What is needed is to shape molecular imaging for problems in regenerative medicine and to apply these tools to small animal and large animal preclinical models, and eventually, patients.
